# A gamepad-like nucleic acid testing device for rapid detection of SARS-CoV-2 via visible nested recombinase polymerase amplification

**DOI:** 10.1038/s44172-024-00229-w

**Published:** 2024-06-21

**Authors:** Ce Liu, Baobao Lin, Guohua Yuan, Zhi Geng, Zhe Zhao, Jiacheng Wang, Jingyu Shao, Zhenqi Wang, Yang Xu, Xujuan Yang, Chang Liu, Yingmei Feng, Xin Fan, Jing Wang, Lili Ren, Yan Xiao, Conghui Wang, Guang Shen, Yi Yang, Chao Zhao, Yinqing Li, Peng Liu, Jingwei Bai

**Affiliations:** 1 School of Pharmaceutical Sciences, Tsinghua University, Beijing, China; 2 Department of Biomedical Engineering, School of Medicine, Tsinghua University, Beijing, China; 3 Beijing Youan Hospital, Capital Medical University, Beijing, China; 4 Department of Infectious Diseases and Clinical Microbiology, Beijing Institute of Respiratory Medicine and Beijing Chao-Yang Hospital, Capital Medical University, Beijing, China; 5 Beijing Key Laboratory of Mental Disorders, National Clinical Research Center for Mental Disorders & National Center for Mental Disorders, Beijing Anding Hospital, Capital Medical University, Beijing, China; 6 NHC Key Laboratory of Systems Biology of Pathogens and Christophe Mérieux Laboratory, Institute of Pathogen Biology, Chinese Academy of Medical Sciences & Peking Union Medical College, Beijing, China; 7 Hangzhou Zijing Biology Inc., Hangzhou, China; 8 Academy of Arts & Design, Tsinghua University, Beijing, China; 9 IDG/McGovern Institute for Brain Research, Tsinghua University, Beijing, China

**Keywords:** Public health, Microfluidics

## Abstract

Nucleic acid tests are essential for the accurate diagnosis and control of infectious diseases. However, current assays are not easily scalable for a large population, due to the requirement of laboratory settings or special equipment. Here, we developed an integrated box for instant nucleic acid screening which fully integrates nucleic acid release, amplification, and results visualization for self-service standalone test. Importantly, the operation of the box runs on a novel gamepad-like interface, which allows deployment of the box in home settings and operation by users without any prior professional training. The performance of the box is empowered by an RNA extraction-free sample inactivation process and nested recombinase polymerase amplification chemistry and exhibits sensitivity comparable to reverse transcription-quantitative polymerase chain reaction with high specificity for severe acute respiratory syndrome coronavirus 2 RNA in a reaction time of 30 minutes directly from fresh swab sample to results. These innovations make the box a novel platform for a convenient, accurate, and deployable point-of-care testing scheme.

## Abstract (web summary)

Ce Liu, Baobao Lin and colleagues report a rapid and straightforward nucleic acid screening test requiring no professional training or formal medical setting. The device runs on a novel gamepad-like interface and fully integrates nucleic acid release, amplification, and visualization.

## Introduction

Emerging viruses have become paramount public health challenges worldwide, including Zika virus, Ebola virus, and most recently severe acute respiratory syndrome coronavirus 2 (SARS-CoV-2), due to its high transmission rate^1–7^. Nucleic acid testing (NAT) of fast sample-to-answer time is essential for monitoring and curbing the virus transmission^8,9^. However, NAT can be vastly overwhelmed by large population size, with NAT laboratory, equipment, and operating personnel shortage as the key bottleneck^10,11^. Point-of-care (POC) NAT holds the potential to alleviate this bottleneck by transforming the landscape of NAT from laboratory-based testing to at-home self-testing^12–14^. Isothermal amplification chemistry, such as Loop-mediated isothermal amplification^15^ and recombinase polymerase amplification (RPA)^16^, combined with CRISPR-mediated detection and fluorescence or lateral flow read-out has enabled the latest development of POC NAT with remarkable sensitivity^17–20^. However, despite these advances, chemistry and procedural complexities remain throughout preparation, reaction, or detection steps, which hinder the full assay integration. Consequently, even the most simplified POC NAT requires additional equipment for heating and liquid handling or personnel with biomedical expertise. Moreover, the risk of leaking amplified samples into the environment further complicates the deployment of these technologies at home^21,22^.

**Fig. 1:**
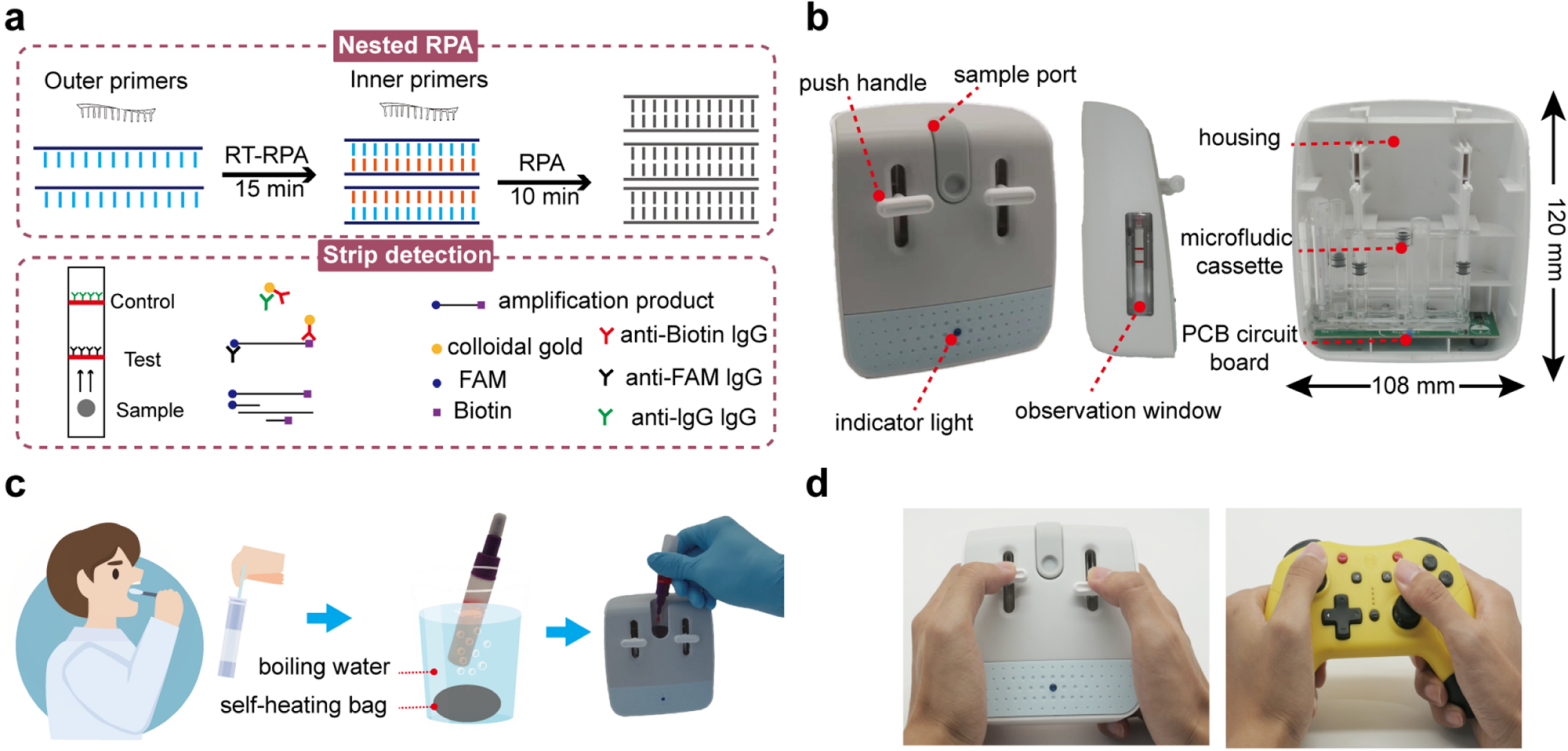
Overview of the box for instant nucleic acid screening (BINAS) system based on nested recombinase polymerase amplification (RPA). **a** The reaction principle and detection process of BINAS. The target nucleic acid is amplified by nested RPA, and then the amplified product is detected by an LFA test strip. **b** Structure of the BINAS device. The device consists of three parts: a PCB heating circuit, a “3D” microfluidic cassette, and the housing (shell). During the detection process, the sample is added to the sample port, the push handle is used to complete the transfer and mixing of the reagents, and the circuit board controls the temperature of the amplification process. **c** Schematic of the BINAS system workflow. The collected samples are heated in the form of a self-heating bag and added to the BINAS for nucleic acid amplification and detection. **d** The designed operation of BINAS is to mimic playing a gamepad to provide a more relaxed user experience while also improving usability.

Here, we report the development of Box for Instant Nucleic Acid Screening (BINAS), a palm-sized, game-pad shaped disposable cartridge, providing swab-to-answer self-NAT at a total cost of $7. Aimed for at-home NAT, BINAS is a fully stand-alone POC NAT technology that streamlines nuclei-acid release, amplification, detection, and lateral-flow strip for direct result visualization in a single 3D microfluidic system (Fig. 1). Operation of BINAS is as simple as sliding buttons, and the entire NAT assay is completely sealed in a one-way flow line of the 3D microfluidic system to prevent leaking upon disposal. BINAS is sensitive, specific, and quick, owing to a newly developed nested RPA chemistry, which achieves limit-of-detection (LOD) of 2 viral copies in less than 30 min. BINAS represents a novel POC platform for complete self-serving NAT by users without any biomedical skill. We envision that BINAS will enable nucleic acid screening in diverse out-of-laboratory scenarios, providing scalable and economical testing at the population level, a critical need for monitoring and controlling any emerging viral transmissions.

## Results

### Nested RPA deployment and improvement

To design a NAT device for swab-to-result test with minimal equipment requirement support, the reaction chemistry must be optimized for steam-lined compatibility, including sample pre-treatment, nucleic acid amplification, and result read-out. RPA was selected as the nucleic acid amplification chemistry, because it can be operated at a moderate temperature range of 37–42 °C achieved by a simple heating board^23,24^. To implement RPA for SARS-CoV-2 virus, we first designed multiple primer sets and probes to specifically target the E, N, and Orf1ab genes of SARS-CoV-2 virus genome and selected primers based on their performance in amplifying standard RNA samples. The best-performing primer sets were found to have a LOD of 100 copies/run, considerably lower than the detection sensitivity of qPCR (Fig. S1).

**Fig. 2:**
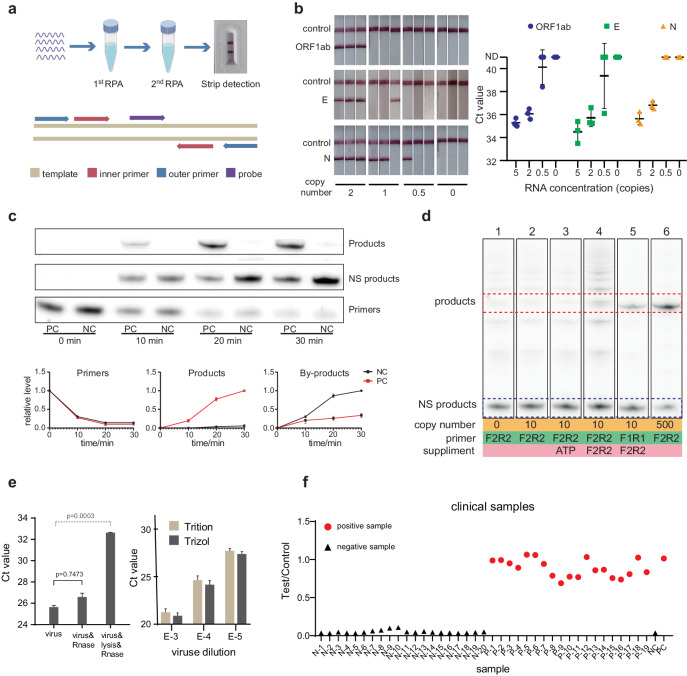
Nested recombinase polymerase amplification (RPA) increased the detection sensitivity for SARS-CoV-2 RNA. **a** Schematic diagram of nested RPA primer design and workflow. RNA samples were amplified by the first RPA with outer primers (indicated by blue arrows) followed by the second RPA with inner primers and probe (indicated by red and purple arrows). **b** LOD for RT-qPCR (right) and nested RPA assay (left). Ct values and LFA results were measured with purified IVD 0–5 copies RNA samples of SARS-CoV-2 E gene, N gene, and ORF1ab gene (*n* = 3 for each assay and the error bar was presented as standard deviation ND indicated Ct value above 45. **c** The dynamics of primers, targeted products and non-specific products (labeled as NS products or by-products in picture) illustrated by polyacrylamide gel electrophoresis at 0, 10, 20 and 30 min of traditional RPA reaction. The quantitative analysis was measured by gray values (*n* = 2). The positive control (PC) experiment is done at 500 copies of target input while negative control (NC) is done at 0 copies of input. **d** The effect of primers and ATP supplements on the amplification result. F1R1 represents the outer forward/reverse primer pair and F2R2 represents the inner forward/reverse primer pair. **e** The efficacy of RNA release by Triton X-100/NP40 lysis buffer on pseudovirus was tested using RNase A aided RT-qPCR assay (left), and a comparison was made between Triton X-100/NP40 lysis buffer and commercialized Trizol reagent on cultured SRAS-CoV-2 (right). The designation “virus & RNase” indicates that the virus sample was directly treated with RNase, while “virus & lysis & RNase” indicates that the virus sample was treated with RNase in lysis buffer containing Triton and NP-40. The dilution ratios of SRAS-CoV-2 culture solution, denoted as “E-3, E-4, E-5,” were 1000, 10,000, and 100,000, respectively. Data are presented in term of the mean ± standard deviation with *n* = 3.

We sought to improve the sensitivity of RPA by utilizing a nested amplification strategy. Nested amplification has been used for gene cloning in traditional PCR, but has been rarely used for NAT, likely due to extended reaction time^25,26^. To design a nested RPA, we selected second sets of primers (inner primer sets) inside the first RPA amplicon. Moreover, an endonuclease-enabled detection probe (inner probe) is designed inside the second RPA amplicon to ensure the sequence-specific detection (Fig. 2a). The experiment began by amplifying the target RNA in the first RPA reaction tube by outer primer sets for 30 min. Subsequently, 1/10 of the products were transferred into the second RPA reaction tube containing fresh reagents and inner primers/probe and reacted for another 30 min. The amplification was monitored either by real-time fluorescence during or a lateral-flow assay following the second RPA step (Fig. 2a). Upon initiating the second RPA, we observed substantial increase in the fluorescent signal, even with an initial input of 2 copies and a 10-min reaction time for the first RPA reaction. This could be attributed to the exponential reaction and signal amplification of the second RPA reaction (Fig. S2a, b).

We then evaluated the LOD of the nested RPA assay in comparison to the US FDA-approved SARS-CoV-2 RT-qPCR assay. Results from RT-qPCR showed a LOD of 2 copies per reaction when targeting three different gene loci, indicating sensitivity of single copy LOD given the variability in diluted input copy numbers (Fig. 2b). The nested RPA exhibited a similar sensitivity for all three genes with LOD of 2 copies per reaction (Fig. 2b). Meanwhile, we confirmed that the improvement in sensitivity cannot be attributed to the shaking of the solution, which had been demonstrated to facilitate the diffusion of amplicon the RPA reaction buffer (Fig. S3). Importantly, in contrast to other two-step detection methods such as DETECTOR or SHERLOCK, nested RPA functions well in the same reaction enzyme and buffer, avoiding any cross-inhibitions between regents for amplification and detection^27,28^. This greatly simplifies reagent formulation, with an additional benefit of economic efficiency. Additional tests were conducted to assess the cross-reactivity of our assay towards other pathogenic microorganisms. The nucleic acid extracts from clinical samples and culture medium of 22 types of commonly encountered respiratory pathogens were used for this purpose. All tests yielded negative results, indicating the high specificity of our assay (Fig. S4).

A common issue with traditional nested amplification techniques is the elongated detection time due to the need for a two-stage amplification. We were surprised to find that nested RPA can detect 10 copies of RNA per reaction in a total of 15 min of amplification time (10 min for the first step and 5 min for the second step, and vice versa, Fig. S5), which is comparable to the recommended reaction time of single-step RPA detection assay. We sought to understand why nested RPA appears to be both more sensitive and faster than single RPA by analyzing the consumption rate of primer and ATP against the generation rate of targeted product during a typical RPA reaction. As illustrated in Fig. 2c, the initial 10 min of reaction generated similar amounts of targeted product and non-specific reaction product, with a substantial consumption (nearly 70%) of primers. In the following 10 min, the generation of targeted product drastically suppressed the non-specific reaction, while the primer concentration further decreased from 30% to 10%. The reaction for both targeted and non-specific reactions appeared to have reached a plateau in the third 10 min, likely due to the low concentration of remaining primers. We also noticed a considerable amount of non-specific reaction product generated in our negative control experiment, suggesting that the non-specific reaction is primarily due to the primer-primer interactions^29,30^. In comparison to the primers, the decrease in the concentration of ATP was much slower (Fig. S6), likely attributed to the ATP regeneration system present in the RPA reagent mixture. Meanwhile, the sensitivity remained unaffected when 1.5–9 mM ATP was incorporated into the RPA reaction system, as illustrated in Fig. S7. Therefore, we propose that the rapid depletion of primers is the primary limiting factor for the generation of targeted products in single-step RPA. However, simply replenishment of the same set of primers or supplementing ATP during the reaction does not increase the total yield of the targeted product, which may be attributed to the competition for primers between non-specific product with targeted product, as they both use the same set of primers. We hypothesize that the inner primers in nested RPA can selectively amplify the desired amplicon from the mixture of amplified products, thereby acting as an efficient relay to boost the amplification of the targeted product. Therefore, we designed a new nested RPA 2.0, in which only the inner primers were added to the first RPA reaction without refreshing the other reagents. Results from Figs. 2d, S8, and S9b demonstrate that the simplified formulation of nested RPA yields a comparable amplification efficiency to the initial version, implying that the addition of the inner primer is all that is necessary for the second RPA. Motivated by this finding, we developed a one-tube nested RPA 2.0 technique to simplify the reaction operation and reduce the reagent cost. This technique involves filling the bottom of the reaction tube with RPA reagent containing outer primer, with the inner primer dried separately on the lid. Upon adding the targeted templates to the reagent at the tube bottom, the first RPA reaction is carried out for 10 min, followed by shaking to mix the inner primer on the lid to initiate the second RPA reaction (Fig. S9a). We evaluated the one-tube nested RPA 2.0 with SARS-CoV-2 RNA sample and found that it had a similar sensitivity with PCR, achieving a LOD of 2 copies RNA of E gene, N gene and ORF1ab gene per reaction (Fig. S10).

In addition, nested RPA2.0 is able to identify multiple targets in one reaction by labeling target-specific probes with different fluorescence or affinity-binding molecules. For SARS-CoV-2 detection with LFA, primers targeting the E gene, N gene and Orf1ab gene were labeled with digoxin, TAMRA, and Biotin, respectively, and these corresponded to three types of capturing antibodies located at different positions on a single LFA test strip (Fig. 1a). Our finding indicated that it is possible to detect pseudo-virus with LOD of 10 copies/reaction for all three genes concurrently. Importantly, when the input copies of the three targets were minimal and their individual detections were susceptible to false negatives (Fig. S11), the multiplex nested RPA 2.0 was still sensitive to produce true positives as long as a single copy of any of the three genes is present in the reaction. The capacity to identify multiple targets simultaneously enables the detection of internal controls in addition to the gene of interests, which is essential to comply with regulatory standards.

Before applying the assay to the COVID-19 clinic sample, we developed a nested RPA 3.0, by combining nested RPA 2.0 with a compatible mixed non-ionic detergent solution as the virus lysing reagent, thus eliminating the need for conventional beads or column purification in addition to upon optimization of amplification chemistry. The lysing process can be conducted at room temperature by incubating the sample with the release reagent for 1 min, and the released sample can be then amplified in the RPA reaction. Our experiment on pseudo-virus demonstrated a high RNA release rate of 98.5%, as measured by a RNase A RT-qPCR assay (Fig. 2e). Additionally, our lysing reagent was found to be comparable to traditional Trizol-based releasing buffer when applied to cultured SARS-CoV-2 virus, as verified by RT-qPCR (Fig. 2e). With this lysis buffer, we were able to detect 10 copies of pseudo-virus using the nested RPA 3.0 (Fig. S12). We then carried out a trial of the entire assay comprising nested RPA 3.0 and LFA read-out on contrived clinical samples. The results precisely matched the clinical record, with 19 positive samples and 20 negative samples identified correctly, indicating 100% sensitivity and specificity on these contrived samples (Fig. 2f).

### Microfluidic device design

**Fig. 3:**
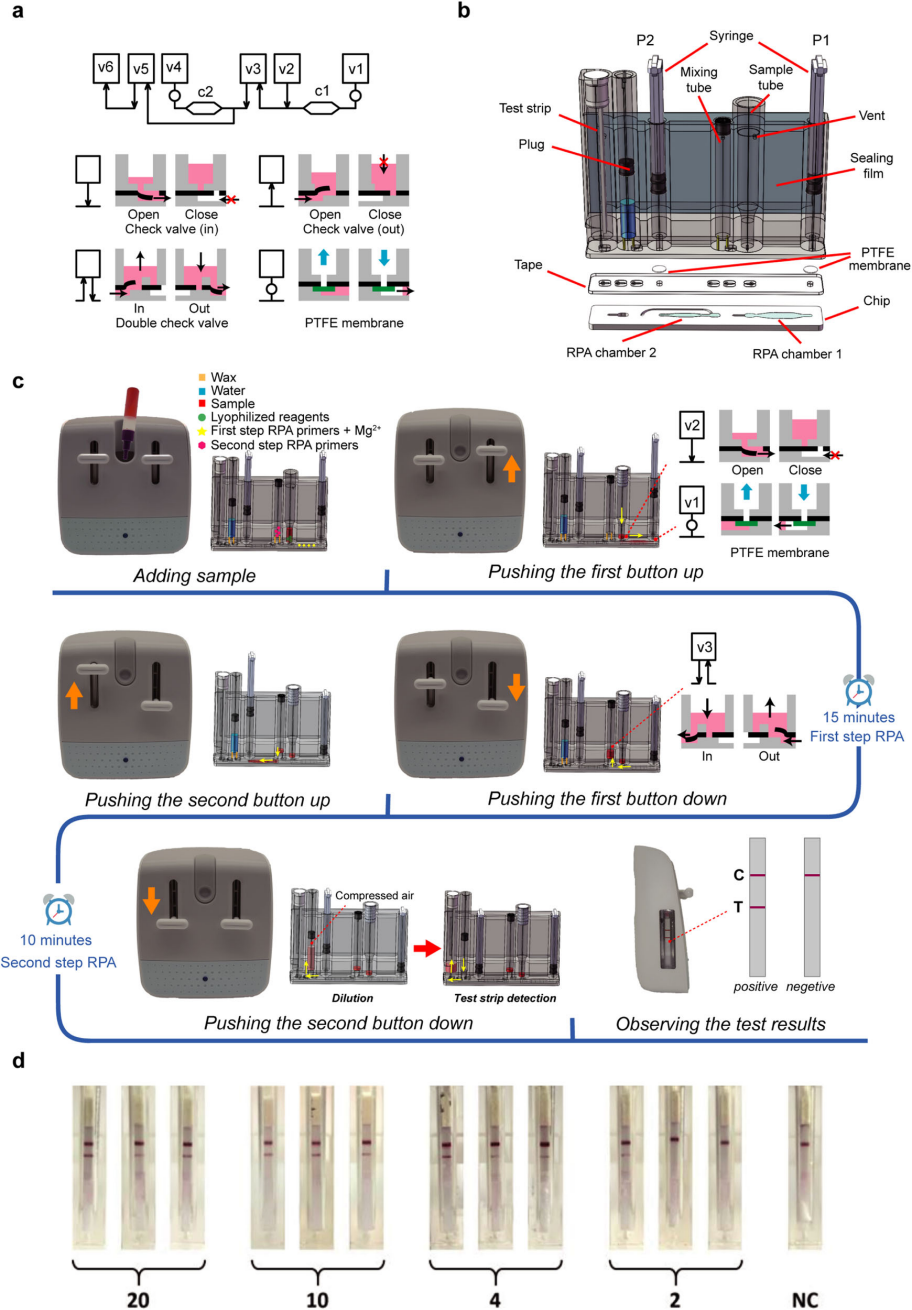
The structure and schematics of the BINAS device. **a** The architecture of the “3D extensible” microfluidic design paradigm (top) and the corresponding basic elements: check valve (in), check valve (out), double-check valve, and precise volume loading unit. **b** The “3D” microfluidic cartridge was made by sandwiching a functional block containing syringe and reagent compartments, a piece of DS tape functioning as the valve, and a one-side carved fluidic chip to precisely control the reaction volume. Reagents and test strips are preloaded in the compartments and sealed by wax for long term storage. Two syringe plungers P1 and P2 provide fluidic driving force. **c** Overview of the reaction workflow: Step 1, adding sample to the sample input port; step 2, pushing up the right button to drive the sample through a one-side valve into the RPA reaction chamber 1 for the first RPA reaction; step 3: after the first RPA is done, pulling the right button down, the amplification product is mixed with inner primer through a double-check valve; step 4, pushing the left button up, the mixture enters the RPA chamber 2 for the second step of RPA amplification; step 5, pulling the left button down to drive the nested RPA amplified products firstly enter the dilution chamber, and due to the air compressing in the dilution chamber, the diluted products is automatically pushed out to the LFA chamber without additional manual operation; step 6, observation of the test results. **d** Detection sensitivity of the BINAS system.

Having established the reaction chemistry, we next sought to develop a stand-alone POC system for self-NAT^31–34^. To this end, we designed a 3D microfluidic cartridge to integrate all the reaction reagents of the nested RPA 3.0, and can complete the liquid aliquoting and transferring through simple operations akin to playing a game, and providing a test strip visualization to indicate the results. This cartridge is composed of three fundamental elements (Fig. 3a), a check valve for the inlet (in), a check valve for the outlet (out), and a double check valve for both the inlet and the outlet (in and out)^35,36^. These microfluidic elements are constructed with a three-layer layout by securing a block and a chip with a double-sided adhesive tape (Fig. 3b). By joining these fundamental elements in sequence, more complicated functional units, for example, sequential loading, and mixing, can be formed to conduct a complex biochemical assay. To ensure accurate aliquoting of the reaction reagents, we incorporated a PTFE (polytetrafluoroethylene) membrane in the block underneath a compartment (V1) to form a precise volume loading unit (Fig. 3a). The hydrophobic nature of the PTFE membrane allows gas to pass through while blocking the movement of aqueous solution. As a result, when the vacuum is applied to the V1, the solution present in the V2 is drawn into chamber C1, which determines the precise reaction volume and is halted in front of the PTFE membrane without any bubble interference. A bubbling-inducing mixing unit is designed to dilute the RPA solution for the lateral-flow assay. This unit works by pressurized injection of the solution through a check valve (in) into a sealed compartment (V5) which is partially filled with the dilution buffer. This injection movement drives a certain volume of air to generate sufficient bubbling turbulence to achieve effective mixing. Once the sealed compartment is sufficiently pressurized, the mixture is then pushed into the second compartment (V6) through the check valve (out) on the other side. This mixing design can achieve mixing and transferring of the solution with a single injection operation. All the driving forces, such as vacuum and pressure, are created by manual actuation through a syringe-like plunger (Fig. 3b), which can be easily operated by hand.

Leveraging the design of these new functional units, we created a schematic diagram that mapped the off-chip operation of nested RPA 3.0 on the 3D microfluidic chip which is composed of a polymethyl methacrylate (PMMA) block with covers, adhesive tape, and a PMMA planar chip (Fig. 3b). The PMMA block is crafted with milliliter-scale compartments to serve as a sample input reservoir, reagent storages and a test strip container, and two polished cylindric compartments are constructed to accommodate plungers to form syringes, thus providing driving forces for liquid movement and bubbling induced mixing. The planar chip is etched with microliter-scale channels and chambers, allowing the accurate aliquoting and transferring of microliter reagents. The block and chip are firmly attached with the help of an acrylic foam-based double-sided tape. A tongue-shaped valve is sculpted on the tape at the junction between compartments and channels/chambers, and is passivated to reduce sticking. An additional low-cost USB-based heating pad is designed and attached to the bottom of the chip to condition nested RPA at its optimal reaction temperature of 37–42 °C (Fig. S13).

Aerosol leakage from any NAT device could lead to potentially long-term experimental contaminations. To prevent aerosol leakage from the cartridge caused by the actuation of the plungers, we designed an air circulation system in the cartridge. Moreover, to avoid any possible leakage of the amplified product, our system includes an air reservoir on the side wall of the cartridge, which is completely sealed with a piece of plastic film and connected to the compartments in the block via air vents. The flexibility of the film allows air to flow between the air reservoir and the compartments, thereby guaranteeing that the cartridge is completely sealed off from the environment once the sample is loaded (Fig. S14).

Lastly, we designed a plastic housing to enclose the cartridge for self-NAT by users without any biomedical skills. The housing resembles a game-pad and transforms the plunger injection into the push-pull of the sliding buttons (Fig. 1b, d). By gamifying the operation process and associating NAT with entertainment, this user interface enhances usability and eliminates negative experiences such as tension and operational difficulties (Fig. S15). We assembled the 3D microfluidic cartridge and the low-cost heating pad with the game-pad-like housing to form the BINAS system. BINAS self-NAT is operated with four simple manual steps. As illustrated in Figs. 3c and S16, a lysed sample solution is first dropped into the sample compartment of the cartridge. This is followed by a manual push of button I to load the lysed sample through the V2 valve into the first RPA reaction chamber C1 for the first round of amplification (step I). This P1-V1-C1 arrangement forms a precise loading unit that specifies the volume of the first RPA reaction. Subsequent pulling of button I dissolves and mixes pre-dried inner primer with the first RPA product in the mixing unit V3 (step II), immediately followed by pushing of button II to transfer the mix into the C2 chamber to initialize the second RPA reaction C2 (step III). Lastly, the pulling of button II dilutes the second-round RPA products in an auto-mixing unit V5 and transfers the dilution to V6 for detection using the lateral-flow assay strip (step IV). BINAS demonstrated a sensitivity of 4 copies of SARS-CoV-2 virus RNA (Fig. 3d), and the whole process took around 24 min.

### Clinical sample testing

**Fig. 4:**
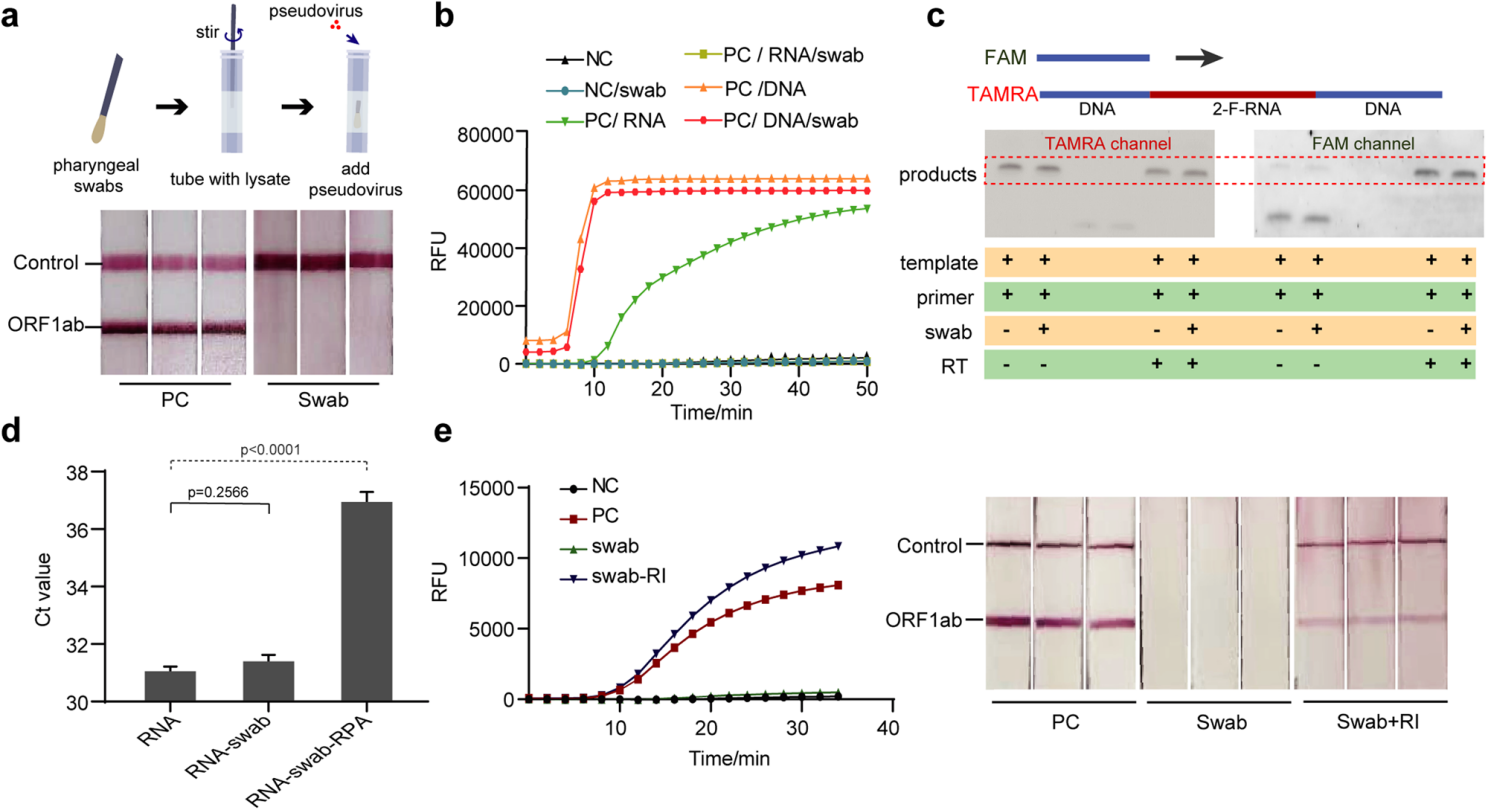
Study of the inhibitory effect on reverse transcription recombinase polymerase amplification (RT-RPA) by fresh swab sample. **a** The experimental workflow (top) and the LFA result indicated the inhibitory effect of the swab sample on RT-RPA reaction (down). The “swab” indicated that pharyngeal swab sample was added into RPA reaction system. **b** Fluorescent RPA assay conducted with various combinations of DNA, RNA, and swab wash solutions. The results demonstrate effective RPA amplification in reactions containing DNA, even in the presence of swabs (labeled as PC/DNA/swab). However, the addition of swab wash solution inhibits amplification in reactions containing RNA (labeled as PC/RNA/swab). This suggests that the inhibitory effect may occur either during the RT reaction step or as a result of RNA degradation. The PC group contained 50 copies of nucleic acid templates, while the NC group did not contain contained samples any nucleic acid templates. **c** The inhibitory effect of fresh swab sample on inhibition RT-RPA using 2-F RNA (2’-Fluoro modificated RNA) template which can serve as normal RT template but is also stable against RNase. The lack of inhibitory effect using 2F-RNA as the RT-RPA template suggests the fresh swab sample did not affect the RT reaction. **d** The degradation of RNA template in different buffers analyzed by RT-qPCR. The Ct value increased dramatically only in the containing swab sample and RPA reagent (labeled as RNA-swab-RPA), indicating that the RNase activity can be strongly enhanced in RPA reaction buffer. Data are presented in term of the mean ± standard deviation with *n* = 3. **e** Inhibiting the RNase activity in swab samples by RNase Inhibitor can restore the RT-RPA amplification, studied by fluorescence (left) and LFA (right) readout assay. In both experiments, 50 copies of pseudovirus containing ORF1ab gene were utilized as the template and the “swab-RI” indicated the RNase inhibitor was added with swab sample in RPA reaction system. Besides, for LFA experiment, pseudovirus containing the Zs-green gene were spiked in as positive control to characterize the validity of the detection results.

It has been reported that directly introducing lysis of human samples without further purification can inhibit PCR or isothermal amplification reactions^37–40^. In our case, we did not observe a inhibitory effect from contrived clinic samples. However, when using freshly taken swab samples, we frequently observed inhibitory effects (Fig. 4a). We set out to investigate the source of this inhibition. We first replaced the RNA with DNA templates and observed no significant loss of LOD, suggesting that the RPA reaction was not adversely affected by the components in fresh swap sample (Fig. 4b). We next used RNase-resistant 2F-RNA as the template and found no significant difference between the fresh swab sample group and control group (Fig. 4c). This result indicates RNA degradation could be the source of the inhibition. Interestingly, we did not detect RNase activity in fresh swab samples mixed with lysis buffer. However, when the mixed sample was introduced to the RPA reaction buffer, a marked increase in RNase activity was observed (Fig. 4d). Subsequent analysis revealed that this type of RNase activity is activated by positive ions of K+, Na+, and Mg^2+^ present in the RPA buffer in a concentration-dependent manner (Fig. S17). Additionally, we discovered that the inclusion of RNase Inhibitor, a recombinant protein that acts as a noncompetitive inhibitor of several RNases, effectively restored the majority of detection signals (see Fig. 4e). This finding indicates that the ion-activated RNase activity present in the RPA buffer was the main cause of the deterioration of LOD when using fresh swab samples (Figs. 4e and S18).

**Fig. 5:**
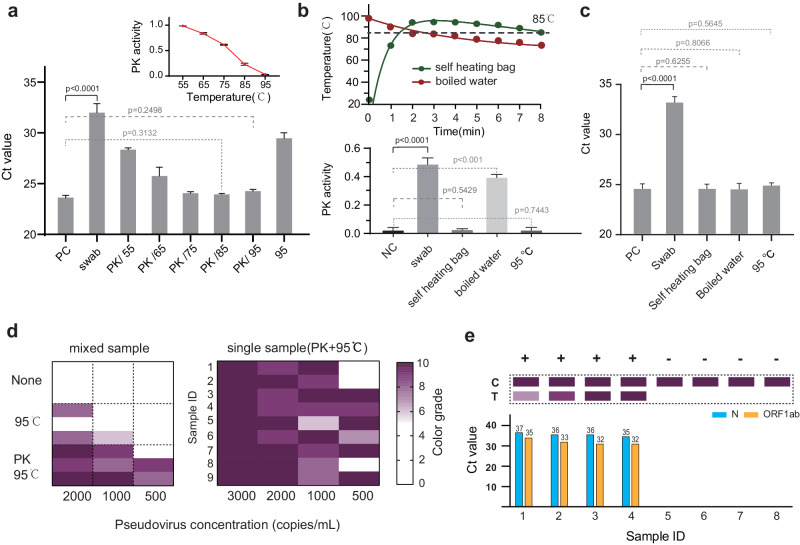
Extraction free sample pre-treatment method enabled by proteinase K (PK) digestion under heating. **a** The RNase activity of fresh swab sample with 5 min treatment of PK at different temperatures, accessed by RT-qPCR measurement of the remaining RNA signal. Inset is the activity of PK at different temperatures. **b** The evaluation of heating condition which is accessible at home: Top the temperature decay curve of the self-heating bag and boiled water; bottom, the activity of PK under different heating condition. **c** The evaluation of RNase activity from fresh swab samples treated by PK with different heating conditions. Data are presented in term of the mean ± standard deviation with *n* = 3. **d** BINAS sensitivity under different sample pretreatment conditions (left). The sample is made of spiking in pseudovirus in mixed swab samples from different donors. In the case of single donor swab sample as the background (right), the PK+ heating can provide a sensitivity of 1000 copies/ml. The color grade is measured according to the color band of the test strip, which is further divided into 11 grades from L0 to L10 (Fig. S17). **e** The performance of extraction-free BINAS (top) on clinic samples with RT-qPCR confirmation (bottom) in which “C” was short for control line and “T” was short for test line.

RNase Inhibitor is expensive and requires stringent storage conditions^41–43^. We next sought to develop a cheaper and more convenient alternative. Proteinase K (PK) digestion in conjunction with heating inactivation is an effective strategy to eliminate RNase^44–46^. This method can be easily implemented by adding the PK to the sample mixture and heating it to boiling. The activity of PK effectively breaks down the RNase before the temperature reaches 85 °C. As the temperature approached the boiling point, the PK was denatured, thus mitigating the risk of further digesting the RPA enzymes (Fig. 5a). Furthermore, we found that heating the fresh swap sample alone without PK was not enough to protect the RNA, likely because of the remaining activity of RNase before it was denatured at high temperature (Figs. 5a and S19). To achieve stable heating conditions outside of a laboratory, we experimented with both boiled water and self-heating bag, which can be easily obtained at home (Fig. 1c). The temperature of boiled water decreased dramatically under 85 °C in 3 min, which was not sufficient to thoroughly inactivate PK (Fig. 5b). In contrast, the self-heating bag was able to rapidly raise the water temperature above 85 °C and maintain it for at least 6 min. Both boiled water and the self-heating bag were successful in driving PK to eliminate RNase activity to a level similar to that of a laboratory-used heating block (Fig. 5c); however, only the self-heating bag was able to completely deactivate the PK (Fig. 5b). By employing PK-heating pretreatment, our BINAS achieved the LOD of 1000 copies per ml in pseudo-virus sample and was able to accurately detect freshly obtained clinical swap sample with qPCR confirmed Ct values up to 37, which is sufficient to detect the majority of cases in the current SARS-CoV-2 pandemic (Figs. 5d, e and S20).

## Discussion

Here we developed one-tube nested RPA technology to amplify and detect the target RNA of SARS-CoV-2. Compared to traditional single-step RPA, this method offers superior sensitivity of nearly single copy per reaction in the same reaction time. By examining the dynamics of substrate consumption, we can conclude that the primers are depleted due to the nonspecific reactions from primer dimers, leading to an unsustainable amplification of target nucleic acid. The implementation of inner primer sets in nested RPA can enhance the sensitivity and specificity of detection by reducing the interference from nonspecific reaction products generated by the outer primers during the initial amplification step. Moreover, the abundance of other substrates, e.g., ATP in the first RPA reaction, ensures the effective reaction throughout the second RPA step. Thus, instead of replacing the entire reaction mix, the nested RPA can be completed by supplementing only the inner primers and probes. This procedure has been simplified by drying the inner primers/probes onto the lid of the first RPA reaction tube, and after the first RPA, the nested reaction can be initiated by inverting the tube and shaking to dissolve the second reaction primer/probes. This One-tube nested RPA has exhibited comparable high sensitivity to the typical two-step nested RPA, thus making it a more cost-effective solution for commercialization products.

To facilitate the two-step operations, we have designed a handheld microfluidic cartridge that integrates all the necessary reactions and detection readout. This cartridge is sealed during the reaction, eliminating the risk of aerosol-based cross-contamination. The device is easy to operate, as the microliter-scale fluid operation in the cartridge can be completed by simply pushing and pulling the rods. This makes the device convenient and quick to use, similar to operating a game handle. Additionally, RPA can be operated at mild reaction temperature (37–42 °C); thus, One-tube nested RPA reactions can be performed in this handheld cartridge with the aid of a PCB-based heating board, with detection sensitivity comparable to that achieved in the laboratory. Moreover, employing industrial injection molding has enabled the mass production of the microfluidic cartridge and casing for the BINAS system. The cost of each microfluidic cartridge, housing, and PCB heating circuit board is just $4. The biological reaction reagents incorporated into the cartridge incur an additional cost of $3. Consequently, the comprehensive swab-to-answer self-NAT procedure can be achieved at a total cost of $7. This cost-effective manufacturing approach underscores the scalability and economic feasibility of the BINAS platform, making it well-suited for widespread deployment and utilization in various settings (Table S2).

A challenge of at-home NAT for COVID-19 is the requirement of sophisticated RNA extraction and purification, for example, column or beads purification, as the inhibitory effect of raw samples can lower the sensitivity of the assay^47–49^. Our study identified that the presence of RNase components in fresh swab samples is the primary cause of inhibition for RT-RPA reaction and that these RNase components can be activated by catalytically Mg^2+^, as well as monovalent ions such as Na^+^ or K^+^. The current PK-heating approach can effectively deactivate the RNase, but further identification of the particular RNase enzyme present in fresh swab samples could aid the development of small molecular inhibitors, resulting in a more straightforward and effective sample pretreatment process. In this study, we focused on the detection of oropharyngeal swab samples within the household setting. Further studies that include the use of nasopharyngeal swabs could improve the reliability of detection for a broader spectrum of applications^50^.

Lastly, self-screening often experiences operational difficulties and induces negative emotions while waiting for the test result. By combining rapid test technology and a narrative-based design approach, BINAS technology has been created to offer a home-oriented application for COVID-19 diagnosis. This technology consists of a simple sample pretreatment, a cartridge with reagents, a PCB heating board and a housing specifically designed for the hand-held operation, all of which work together to provide an engaging user experience. The design of BINAS replaces the professional operation with a push-pull handle used in gaming, thus providing a more relaxed user experience while also improving usability. By leveraging nested RPA technology, sample-to-answer time was reduced to 30 min, with the addition of positive interference during the waiting period to improve user experience. Compared to existing home NAT products, BINAS demonstrates comparable sensitivity and speed, while maintaining a lower cost. Through extensive trials during the COVID-19 outbreak, BINAS was highly recognized by users for its pleasantness, ease of operation, and rapid result visualization.

## Methods

### Virus and clinical samples

The SARS-CoV-2 pseudovirus and RNA used in this study were purchased from the National Standard Material Resource Sharing Platform with the product number: NIM-RM5203 and GBW(E)091099, respectively. The DNA fragments of SARS-CoV-2 E gene, N gene and ORF1ab gene were synthesized by Beijing Hongxiang Biotechnology Co., Ltd and were cloned into pUC57 vectors with T7 promoter. The RNA samples were prepared by in vitro transcription of the above gene using T7 RNA polymerase. The clinical samples related to COVID-19 used in this study were from Beijing Ditan Hospital and Beijing Youan Hospital. The cultured SARS-CoV-2 was obtained from Institute of Pathogenic Microbiology, Peking Union Medical College. Clinical simulation samples were obtained from healthy volunteers. The clinical samples or microorganisms used for cross-reaction testing were obtained from the Beijing Chaoyang Hospital with informed consent.

### The operation of RPA and nested RPA

The RT-RPA kits were purchased from Weifang Amp-Future Biotech Co., Ltd. Primers and probes targeting the E gene, N gene, and ORF1ab gene of SARS-CoV-2 were designed following the protocol of Twist-Dx (Maidenhead, UK), and the sequences tested are listed in Table S1. The RT-RPA reactions were performed following the manufacturers’ protocol. Briefly, 19.4 μl buffer A, 2 μl forward primer, 2 μl reverse primer, 0.6 μl probe, 1 μl RNA template, 13.5 μl deionized water, and 2.5 μl buffer B were mixed with RT-RPA mix enzymes powder. The above solution was heated with a constant temperature metal-bath at 42 °C for 15 min. Five μl of the reaction products were diluted with 100 μl DI water, and the dilution was tested with a colloidal gold lateral flow assay strip which can be read out in 5 min.

For the nested RPA reaction, 5 μl products from the first RPA reaction (19.4 μl buffer A, 2 μl RPA-1F, 2 μl RPA-1R, 1 μl RNA template, 14.1 μl deionized water and 2.5 μl buffer B) were added into the second RPA reaction (19.4 μl buffer A, 2 μl RPA-2F, 2 μl RPA-2R, 0.6 μl probe, 1 μl RPA template, 13.5 μl deionized water and 2.5 μl buffer B) for another 10 min amplification at 42 °C with metal bath followed by LFA assay as to standard RPA.

The nested RPA 2.0 was similar to nested RPA in principle and reagent except that the first RPA reaction system (template, primers, and RPA reagent) and the second PRA primers and probe were loaded on the bottom and cap of the reaction tube respectively. Upon detection reaction, RNA template was amplified with the first RPA reaction on the bottom of the tube for 10 min at 42 °C followed by the addition of the second RPA primers and probe on the cap by shaking. After another 10 min reaction at 42 °C the products were tested with an LFA strip as described above.

The operation procedure of fluorescent RPA and nested RPA 2.0 were slightly different from the LFA counterpart. The signal of fluorescent RPA was monitored on qPCR instrument CFX-96 (Bio-Rad) every 2 min during the reaction. For nested RPA 2.0, the signal was only monitored during the second RPA reaction.

For multi-target nested RPA detection, RNA templates were amplified in the first RPA reaction with primer sets targeting the E gene, N gene, and ORF1ab gene at a concentration of 0.4 μM, respectively, followed by the second RPA reaction with primers and probes labeled with biotin, digoxin and TAMARA, respectively. The LFA strip for this work was preloaded with anti-biotin, anti-dig, and anti-TAMRA antibodies on the selected detection area.

### RT-qPCR for reference study

The extracted virus RNA containing target genes was detected with a one-step RT-qPCR kit (NEB) according to the manufacturer’s instruction. Briefly, 10 μl reaction buffer was mixed with 0.8 μl forward primer, 0.8 μl reverse primer, 0.4 μl Taqman probe, 1 μl RNA sample, 1 μl enzyme mix, and 6 μl DI water. The reaction was performed using a qPCR instrument CFX-96 (Bio-Rad), with the following program: reverse transcription at 50 °C for 10 min, pre-denaturation at 95 °C for 3 min, and followed by 45 cycles of denaturation at 95 °C for 10 seconds and annealing and extension at 55 °C for 30 s. All primers used in the RT-qPCR assay were listed in Table S1.

### Measurement of the ATP and primer consumption during the RPA reaction

Samples of 2 μl volume were collected at different time-point from a 50 μl standard RT-RPA reaction, and measured through a chemiluminescence assay according to the manufacturers’ protocol (Beyotime, China). For primer-consuming measurement, 12% denatured PAGE gel was prepared with 8 M urea. RT-RPA reaction was performed following standard RT-RPA protocol in which 500 copies (PC) or 0 copy (NC) SARS-CoV-2 RNA was amplified with primers targeting E gene that labeled by TAMRA fluorescein on the 5’ end of the reverse primer. Two μl volume of products were taken out at different time points (0, 10, 20 and 30 min) during the reaction and were placed immediately in liquid nitrogen for termination. Collected samples were mixed with 5 μl RNA loading buffer (Thermo Fisher) and 3 μl DI water followed by heating at 98 °C for 5 min and subjected to. PAGE gel electrophoresis at 150 V for 30 min for nucleic acid separation. Finally, TARMA labeled products and primers on the gel were detected by fluorescence imager (Typhoon, General Electric).

### Measurement of the RNase activity from throat swab sample

The RNase-probe with a sequence of 5’-TAMRA-UUUUUUUU-BHQ2-3’ was chemically synthesized by Beijing Rubio Biotech Co., Ltd. To measure the RNase activity, a fresh throat swab sample taken from healthy donor was dissolved in 1 ml lysis buffer (10 mM Tris-HCl pH 8.0, 0.25% Triton X-100 and 0.25% NP40) and centrifugation at 12,000 × *g* for 1 min. Then, 10 μl of the supernatant was mixed with 1 μl of 10 μM RNase probe and 1 μl of 1 M potassium acetate solution, followed by incubating at 42 °C for 2 min. The above solution was then diluted with 90 μl of deionized water, and the fluorescence intensity was measured by a microplate reader (Tecan Spark).

### Determine the activity of proteinase K

The proteinase K digestion assay was prepared containing 50 μl PBS solution, 0.1 μg EGFP, and 0.1 μg PK, with EGFP in DI as the NTC (no template control) and PK in DI as the negative control (NC). The change of fluorescence intensity was accessed by qPCR instrument every 2 min for a total of 20 cycles at 55 °C. The relative activity of PK was calculated as follows: *i* = RFU_test_/(RFU_NC_ − RFU_NTC_), in which RFU_test_, RFU_NC_, and RFU_NTC_ was represented for the relative fluorescent unit of the test group, NC and NTC, respectively.

### Lysis assay to release the viral RNA

The lysis buffer in this study was composed of Tris-HCl (10 mM, pH 8.0), 0.25% Triton X-100, and 0.25% NP40. For initial evaluation of the lysis capability, pseudovirus was incubated for 1 min in the above lysis buffer for the test group or in 10 mM Tris-HCl buffer for the control group. The solution was then digestion with RNase at 1 ng/μl concentration. The remaining RNA from the still intact viral particle were extracted by magnetic beads followed by RT-qPCR assay to measure the Ct value. The release rate was calculated by comparing the Ct values of the lysis group and the control group.

To evaluate our lysis assay in the case of native SARS-CoV-2 virus, viral culture was firstly subjected to 10 times gradient dilution from 10^−3^ to 10^−5^. Each dilution was treated with our lysis buffer vs standard Trizol reagent for 1 min at room temperature. Then, samples treated with Trizol were purified by magnetic beads before the RT-qPCR, and samples treated with our lysis buffer were directed to RT-qPCR without any further treatment. The lysis efficiency was calculated by comparing the Ct values of the two groups.

### Sample pretreatment by PK with heating

To mimic the clinical condition, we spiked different concentrations of pseudovirus in throat swab samples taken from healthy donors. These samples were treated with 50 μg/ml PK at 95 °C for 5 min, and subjected to nested-RPA detection directly without RNA extraction. To exert the heating treatment at home environment, the sample tubes were treated in a 500 ml water bath heated by a 100 g self-heating bag for 5 min.

### The fabrication of the microfluidic device

The microfluidic cassette was made of PMMA by injection molding method. PTFE membranes (Shanghai Yifang Biotechnology Co., Ltd.) were cut into disks with a diameter of 4.5 mm using a puncher. The non-adhesive patterns on both sides of the double-sided tape (DS tape) were achieved as follows (Fig. S21): first, die-cutting knives which have the non-adhesive patterns were first fabricated using milling and drilling techniques. Then, the release paper (CY9970, Yichuang Electric, Suzhou, China) was punched using the die-cutting knives to form masks. A piece of DS tape was covered with the patterned release paper from both sides and holes were punched on the tape. Next, talcum powders were evenly smeared on the surfaces of the release paper. The exposed DS tape can trap the talcum powders to lose its adhesion and the excess talcum powders were blown away by nitrogen. Finally, with the aid of an alignment tool, the upper and the lower PMMA blocks were bonded together using the double-sided tape. After bonding, the microfluidic cassette was stored at room temperature for 48 h to maximize the bonding strength. The BINAS box contains of a microfluidic device and a printed circuit board, which functions as a heater for the amplification reaction. The box is made of ABS and is fabricated using plastic injection molding. All the rubber stoppers were injection molded with butyl rubber.

### Self-operation of BINAS from sample to answer

During the detection process, a collected throat swab is initially inserted into a provided sample tube. This tube includes a developed lysis solution (Tris-HCl, 10 mM, pH 8.0, 0.25% Triton X-100, and 0.25% NP40). Additionally, PK is added to eliminate nucleases present in the human sample. Subsequently, the sample tube is heated for 10 min using a self-heating bag. After that, the processed sample was transferred into the sample tank of BINAS and was sealed with a rubber stopper. Next, connect the BINAS to the power supply, and manually push the push handle 1 up to carry out the first step of RPA amplification for 15 min. At this time, the indicator light is in a constant state. After the reaction is completed, the indicator light flashes. Then, push down the push handle 1 to mix the product of the first step of RPA amplification, and then push up the push handle 2 to carry out the second step of RPA amplification for 10 min. At this time, the indicator light is in a constant state. After the reaction is completed, the indicator light flashes. Finally, push the push handle 2 downward, and the second step RPA amplification products enter the detection tank after being diluted. After waiting for 5 min, check the results of the test strips.

### Statistics and reproducibility

The Ct value data obtained in this study were analyzed with GraphPad Prism 8 (GraphPad Inc., USA), and the image data was analyzed using Image J (2.9.0/1.53t). The group variables were evaluated using Student’s *t* test (two-sided) with at least three biological duplications. The values with *p* < 0.05 were considered significant.

### Reporting summary

Further information on research design is available in the Nature Portfolio Reporting Summary linked to this article.

### Supplementary information

Supplementary Material

Reporting Summary

## Data Availability

The data that support the findings of this study are available from the corresponding author upon reasonable request.
