# Supplementary material for: A gamepad-like nucleic acid testing device for rapid detection of SARS-CoV-2 via visible nested recombinase polymerase amplification
Source: Commun Eng. 2024 Jun 21;3:83. doi: 10.1038/s44172-024-00229-w (PMC11192882; doi:10.1038/s44172-024-00229-w)
Supplement: Supplementary file 1 — Supplementary Material [file 44172_2024_229_MOESM1_ESM.pdf]

Supplementary materials for

A gamepad-like nucleic acid testing device for rapid detection of SARS-CoV-2 via visible nested recombinase polymerase amplification

This supplementary PDF file includes the following information:

- Table S1. The sequence of primers used in this study.
- Table S2. A comparative analysis of COVID-19 products for home nucleic acid testing
- Figure S1. LOD assay of standard RPA in SARS-Cov-2 RNA detection.
- Figure S2. The detection sensitivity of SARS-Cov-2 was improved by nested RPA
- Figure S3. Comparison of nested RPA and standard RPA with shaking
- Figure S4. Cross-reaction assessment of nested RPA towards other pathogenic microorganisms.
- Figure S5. Detection time testing of nested-RPA technology with sensitivity of 10 copies per reaction.
- Figure S6. Detection of changes in ATP concentration during RPA reaction.
- Figure S7. The effect of adding ATP in RPA detection sensitivity
- Figure S8 The quantify assay of the improvement of the second primer set in nested RPA
- Figure S9. The principle and workflow of nested RPA 2.0.
- Figure S10. LOD assay of nested RPA 2.0 for SARS-Cov2 E gene, N gene and ORF1ab gene.
- Figure S11. multiple targets detection in on reaction by nested RPA 2.0.
- Figure S12. Lysis buffer improves detection sensitivity for virus by nucleic acid release.
- Figure S13. Cartridge heating system.
- Figure S14. The air circulation system in the cartridge.
- Figure S15. Thumb thrust test on 10 volunteers under relaxed conditions.
- Figure S16. Overall BINAS workflow.
- Figure S17 RNase activity is activated by positive ions in a concentration-dependent manner.
- Figure S18. RNase Inhibitor was able to restore most detection signal.
- Figure S19. RNase was totally digested by PK at high temperature.
- Figure S20. Detection of simulated samples and clinical samples.

- Figure S21. Production of double-sided tape.

Table S1. The sequence of primers used in this study

Primers	Sequence (5'-3')
E-RPA 1F	ATGTACTCATTCGTTTCGGAAGAGACAGG
E-RPA 1R	AGACCAGAAGATCAGGAACT CTAGAAGAA
E-RPA 2F	TTCTTTTTCTTGCTTTCGTGGTATTCTTGC
Bio-E-RPA 2R	Biotin-AAGAAGGTTTTACAAGACTCACGTTAACAAT
TAMRA-E-RPA 2R	TAMRA-AAGAAGGTTTTACAAGACTCACGTTAACAAT
Dig-E-RPA 2R	Dig-AAGAAGGTTTTACAAGACTCACGTTAACAAT
E-RPA probe（Nfo）	FAM-TTACACTAGCCATCCTTACTGCGCTTCGAT [THF]GTGTGCGTACTGCTG-C3 spacer
E-RPA probe（Exo）	TTACACTAGCCATCCTTACTGCGCTTCGAT-FAM-[THF]GT-BHQ1-GTGCGTACTGCTG-C3 spacer
E-qPCR F	ACAGGTACGTTAATAGTTAATAGCGT
E-qPCR R	ATATTGCAGCAGTACGCACACA
E-qPCR probe	FAM-ACACTAGCCATCCTTACTGCGCTTCG-BHQ1
N-RPA 1F	TTCCTCATCACGTAGTCGCAACAGTTCAAG
N-RPA 1R	CTTAGAAGCCTCAGCAGCAGATTTCTTAGTG
N-RPA 2F	AAGAAATTCAACTCCAGGCAGCAGTAGGGG
Biotin-N-RPA 2R	Bio-ACAGTTTGGCCTTGTTGTTGTTGGCCTTTA
Dig-N-RPA 2R	Dig-ACAGTTTGGCCTTGTTGTTGTTGGCCTTTA
TMARA-N-RPA 2R	TMARA-ACAGTTTGGCCTTGTTGTTGTTGGCCTTTA
N-RPA probe	FAM-AACTTCTCCTGCTAGAATGGCTGGCAATGG [THF]GGTGATGCTGCTCTTGC-C3 spacer
O-RPA 1F	TGTAGTTGTGATCAACTCCGCGAACCCATGCT
O-RPA 1R	TGTAGTTGTGATCAACTCCGCGAACCCATGCT
O-RPA 2F	TCAGTCAGCTGATGCACAATCGTTTTTAAACG
Bio-O-RPA 2R	Bio-CTTGGAAGCGACAACAATTAGTTTTTAGGA
TAMRA-O-RPA 2R	TAMRA-CTTGGAAGCGACAACAATTAGTTTTTAGGA
Dig-O-RPA 2R	Dig-CTTGGAAGCGACAACAATTAGTTTTTAGGA
O-RPA probe（Nfo）	FAM-AGCCCGTCTTACACCGTGCGGCACAGGCAC T/THF/GTACTGATGTCGTAT-C3 spacer
O-RPA probe（Exo）	AGCCCGTCTTACACCGTGCGGCACAGGCAC T-FAM/THF/GT-BHQ1ACTGATGTCGTAT-C3 spacer
O-qPCR F	TGTGATCAACTCCGCG
O-qPCR R	TTGTGATCAACTCCGCGAACCC
O-qPCR probe	FAM-AGCCCGTCTTACACCGTGCG-BHQ1
2F-RNA Template	TAMRA-AGTGCCTGTG-i2FC-i2FC-i2FG-i2FC-i2FA-i2FC-i2FG-i2FG-i2FU-i2FG-TAAGACGGGCTGCA CTTAC

2F-RNA primer	FAM-GTAAGTGCAGCCCGTCTTA

Table S2. A comparative analysis of COVID-19 products for home nucleic acid testing

Product	Type of NAT	Price	Sensitivity	Time
**Lucira**	Isothermal	$35	2700copies/ml	30mins
**Cue Health**	Isothermal	$33	/	20mins
**Visby Medical**	RT-PCR	$50	/	30mins
**BINAS**	Isothermal	$7	1000copies/mL	30mins

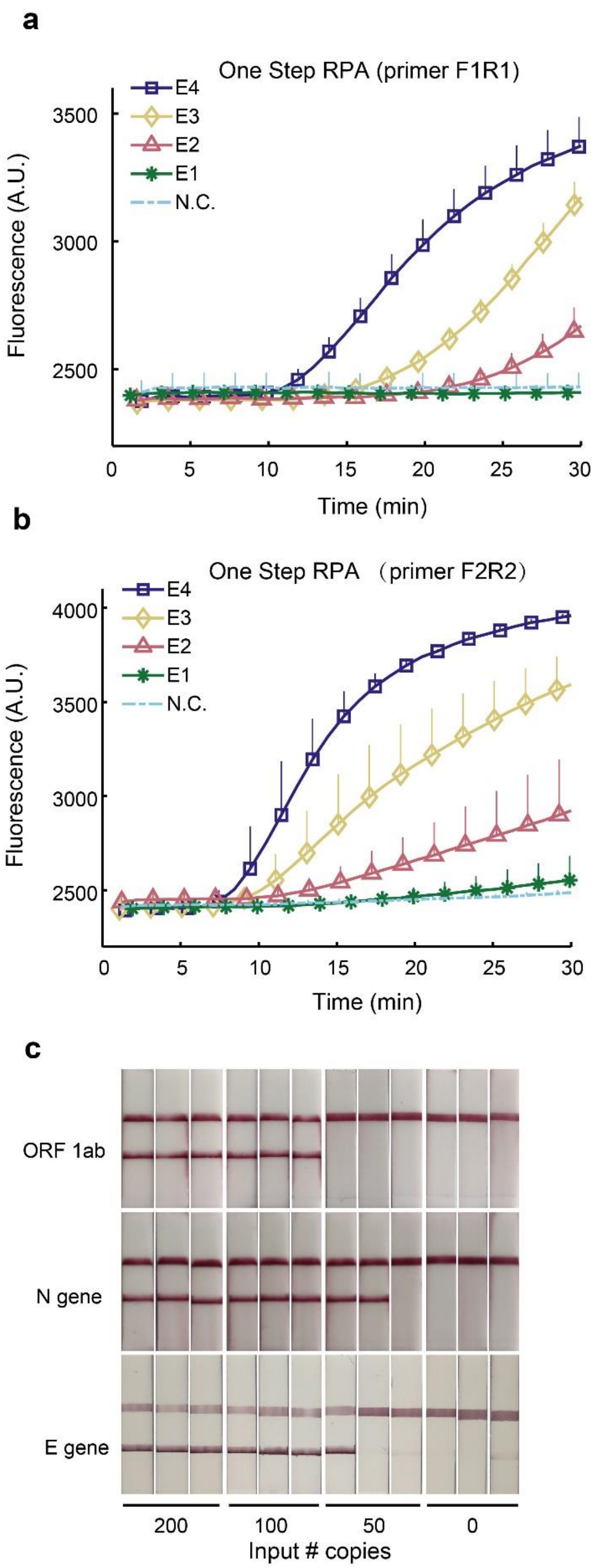

Figure S1. LOD assay of standard RPA in SARS-Cov-2 RNA detection.

a-b) Fluorescence RPA amplification was performed with two pairs of primer sets using a gradient diluted RNA template (SARS-Cov-2 E gene) and the products were monitored as fluorescence curves. As shown in a-b, only samples with virus above 100 copies can be detected as positive indicating the LOD of standard Fluorescence RPA was 100 copies per reaction (the reaction volume was 50ul). Data are presented in term of the mean ± standard deviation with n=3. c) LOD testing of standard RPA with LFA for SARS-Cov-2 RNA detection. RNA targeting three SARS-Cov-2 genes were used as templates to determine the sensitivity of RPA technique coupled with LFA. The copy number used in this test was labeled on the bottom of the picture

where the low band represents positive result indicating the detection line of RPA-LFA was 100 copies per reaction (the reaction volume was 50ul).

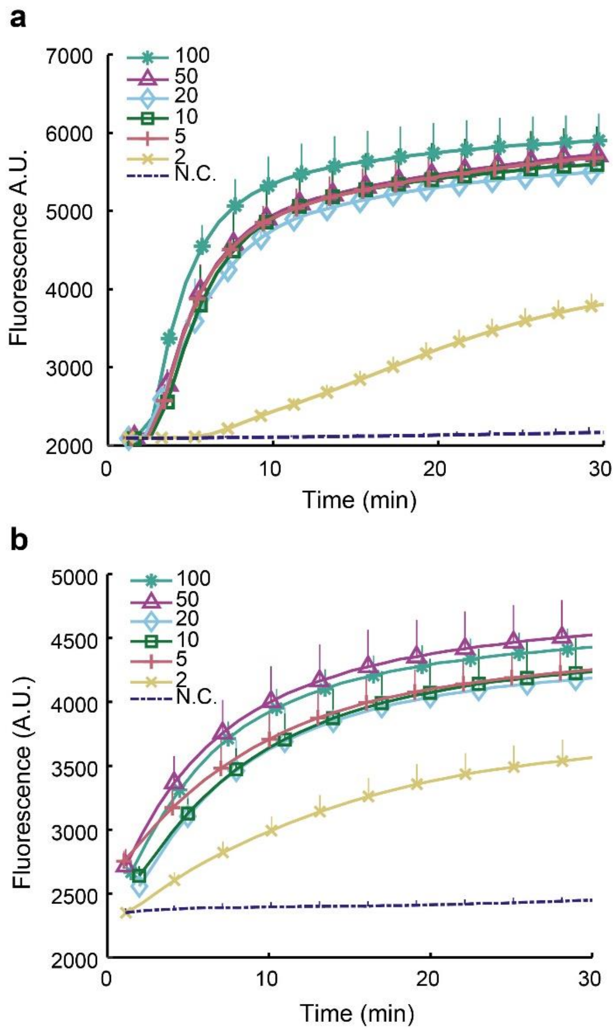

Figure S2. The detection sensitivity of SARS-Cov-2 was improved by nested RPA.

Gradient diluted RNA (the copies number was labeled on the legend) templates were subjected to the first RT-RPA reaction with outer primers (E-RPA 1F and E-RPA 1R) for 30 (a) or 10 (b) min respectively followed by second RPA reaction with inner primers (E-RPA 2F and E-RPA-2R) and fluorescence probe (E-RPA probe). The intensity of fluorescence was monitored for 30 min. As shown in a-b, the fluorescent signal was immediately increased upon the second RPA reaction and the detection line of nested RPA was 2 copies per reaction in both cases (first RPA for 10 min or 30 min). Data are presented in term of the mean ± standard deviation with n=3

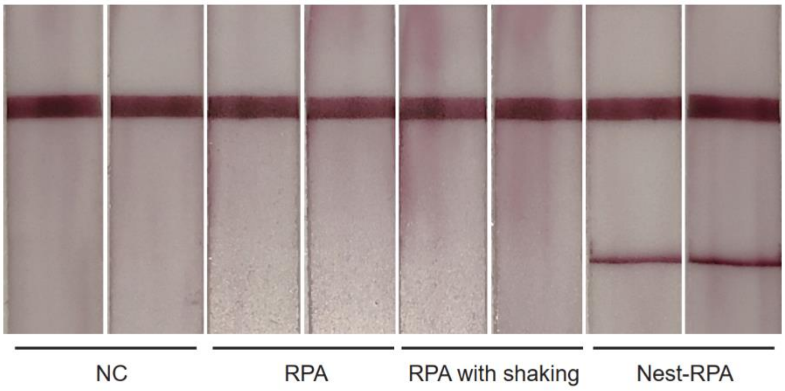

Figure S3. Comparison of nested RPA and standard RPA with shaking

2 copies of IVD RNA samples were subjected to RPA reaction for 10 min at 42℃. For the 'RPA with shaking' group, the reaction tubes were shaken vigorously for 10s followed by another 10-minute reaction. For the 'nested RPA' group, outer primers set were added and mixed sufficiently followed by another 10 min reaction. No RNA sample was added into the NC group.

S/N	species	source	result	S/N	species	source	result
D2	Streptococcus pneumoniae	Clone	-	1	mycobacterium tuberculosis	Clinic	-
D4	Candida glabrata	Clone	-	2	Cryptococcus neoformans	Clone	-
T3	Candida albicans	Clone	-	5	aspergillus fumigatus	Clone	-
D5	Klebsiella pneumoniae	Clinic	-	11	Streptococcus pyogenes	Clone	-
T10	Influenza A virus	Clinic	-	14	Haemophilus influenzae	Clone	-
T7	Mycoplasma pneumoniae	Clinic	-	15	Cytomegalovirus, Mycoplasma pneumoniae	Clinic	-
D1	Staphylococcus aureus	Clone	-	16	Legionella pneumophila	Clinic	-
T6	Human Respiratory Syncytial Virus	Clone	-	17	Rhinovirus human metapneumovirus	Clinic	-
T11	H1N1, RS virus	Clinic	-	18	Influenza B virus	Clinic	-
T9	Legionella pneumophila	Clinic	-	19	Cytomegalovirus, EB virus		-
T13	Adenoviridae	Clinic	-	20	Cytomegalovirus		-
NC	H_2O		-	NC	H_2O		-
PC	SARS-CoV-2		+	PC	SARS-CoV-2		+

Figure S4. Cross-reaction assessment of nested RPA towards other pathogenic microorganisms.
22 types of commonly encountered respiratory pathogens samples were detected with nested RPA procedure using primers targeting the E gene, N gene and ORF1ab gene. These samples were from clinic situation or pure culture that labeled with "clinic" and "clone" respectively. All samples except PC (pure SARS-Cov-2 RNA) showed negative results in this test indicating the high specificity of nested RPA technology in nucleic acid detection.

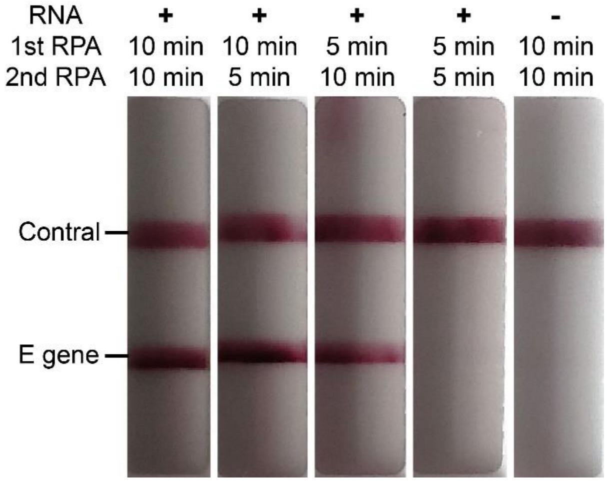

Figure S5. Detection time determination for nested RPA technology in LOD of 10 copies per reaction.
10 copies of SARS-Cov-2 RNA targeting E gene were subjected to nested RPA procedure with different first and second reaction time labeled on top of the picture. The positive results were shown on LFA when the reaction time was 10 minutes for first step and 5 minutes for the second step, and vice versa, indicating the detection time of nested RPA for 10 copies per reaction was less than 15 min.

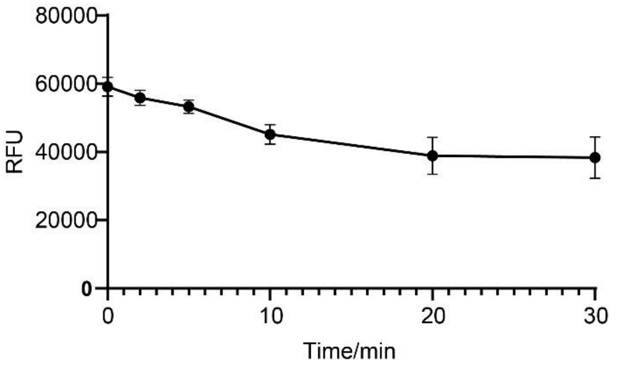

Figure S6. Detection of changes in ATP concentration during RPA reaction.

500 copies of virus RNA was amplified with RPA reaction for 30 min. The content of ATP in reaction buffer was evaluated by luciferase method at different time points (0min, 2min, 5min, 10min, 20min and 30min). The x-axis represents reaction time and the y-axis represents the intensity of chemiluminescence emitted by luciferase taking ATP as substrate. Data are presented in term of the mean ± standard deviation with n=3

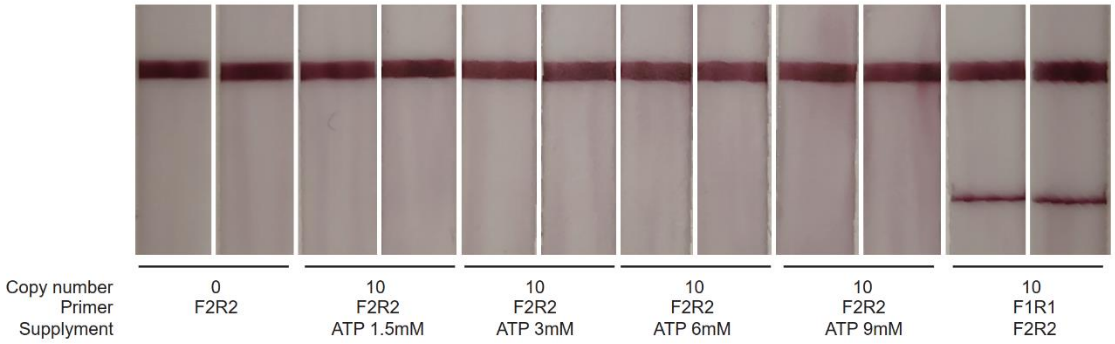

Figure S7. The effect of adding ATP in RPA detection sensitivity

10 copies of IVD RNA samples were subjected to RPA reaction with different concentrations of ATP addition. The NC group was reaction with no RNA, and the PC group was nested RPA reaction with 10 copies of RNA.

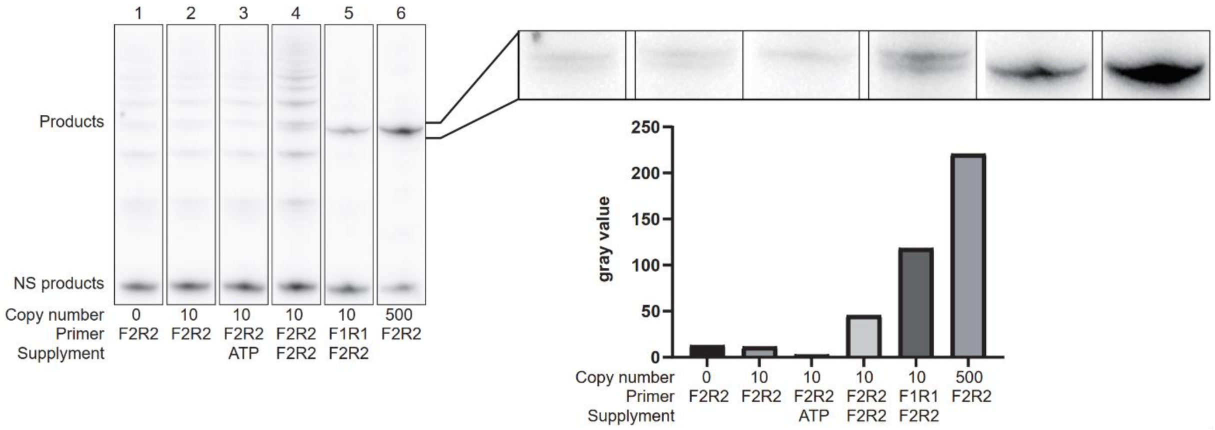

Figure S8 The quantify assay of the improvement of the second primer set in nested RPA.

The products area in Fig.2d was partially enlarged to show the band pattern clearly. The band in the products area was evaluated by gray value calculation and the results were demonstrated by histogram (below).

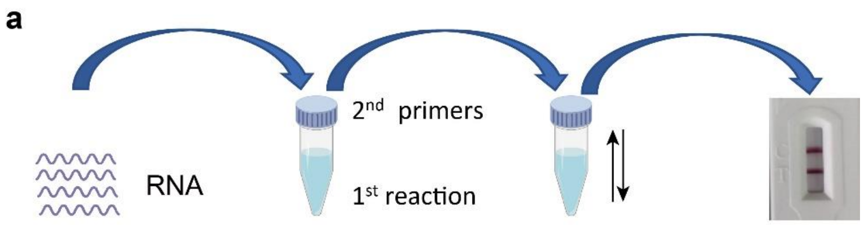

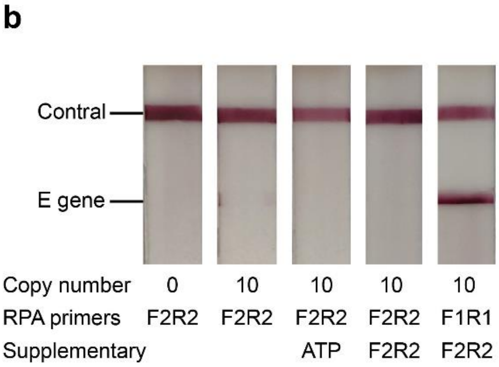

Figure S9. The principle and workflow of nested RPA 2.0.

a). The workflow of nested RPA 2.0. The outer primers and probe fixed on the cap tube were added into second RPA reaction by shaking following 10 min's first RT-RPA reaction. The final products were determined by LFA. b). Standard RPA was performed with inner (F2R2) or outer (F1R1) primers for 10 min followed by supplementing of primers (inner or outer primers) or ATP for continued reaction. The results were shown by LFA strip where only the sample performed in reaction with outer primer for first RPA followed by addition of inner primer for next step amplification exhibit positive line in LFA.

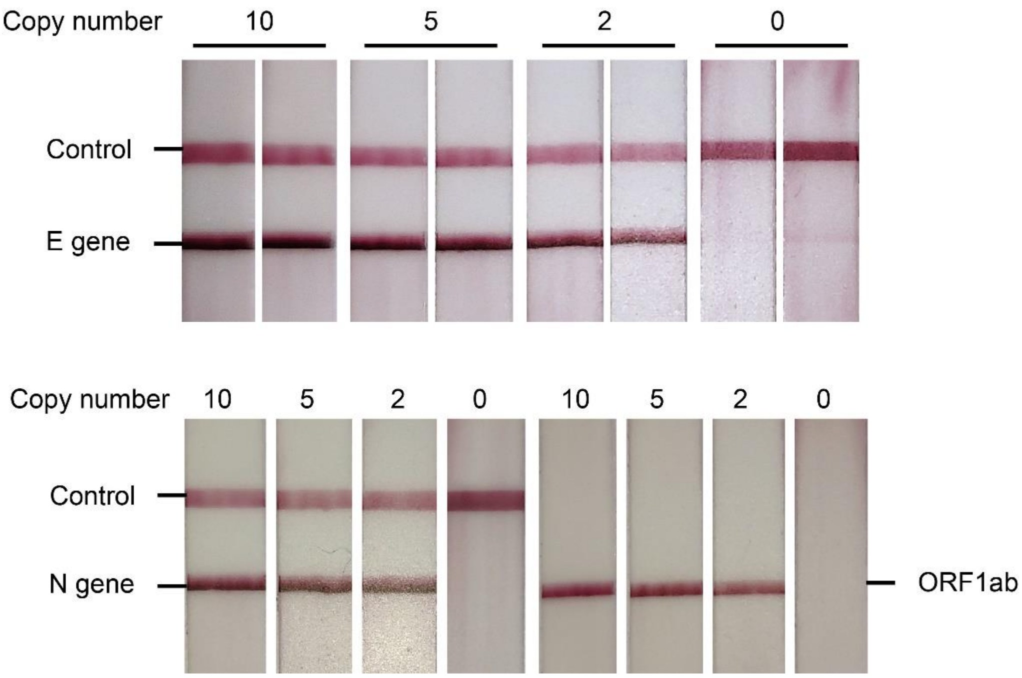

Figure S10. LOD assay of nested RPA 2.0 for SARS-Cov2 E gene, N gene and ORF1ab gene.

The LOD of nested RPA 2.0 was evaluated with gradient diluted RNA as templates in which the copy number was labeled on top of each independent picture. For each of three targets, the LOD of nested RPA was 2 copies of RNA per reaction. The time consume for this test was 20 min (10 min for the first RPA reaction plus 10 min for the second RPA reaction). The LFA strips used in ORF1ab detection were labeled with

antibodies for three targets (biotin, digoxin and TAMRA) only for which reason the control line did not appear.

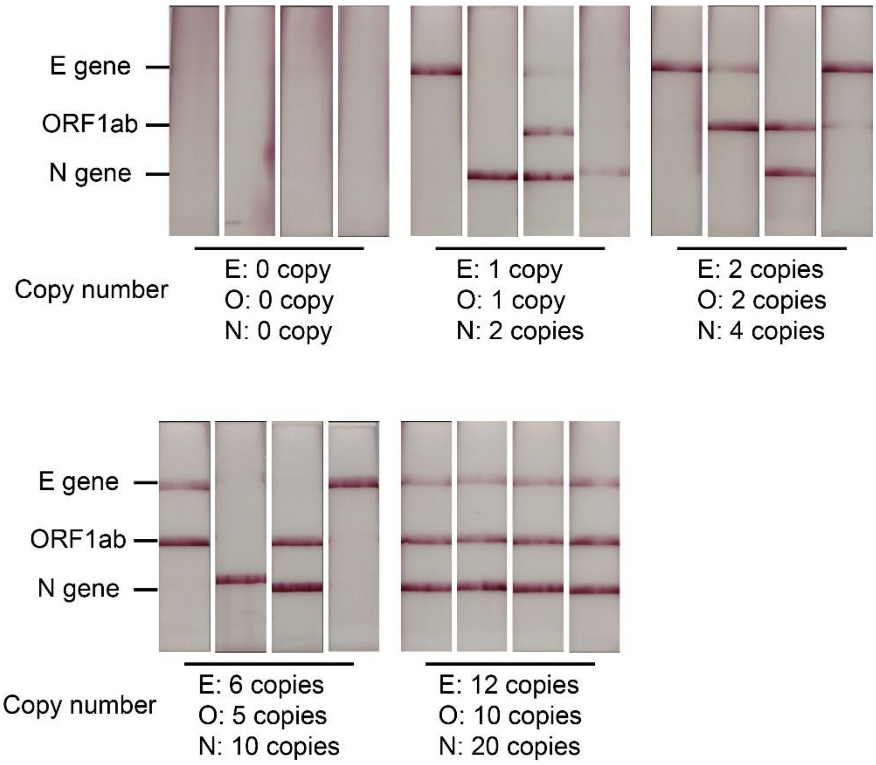

Figure S11. Multiple targets detection by nested RPA 2.0.

The multiple nested RPA 2.0 was performed with the first RT-RPA primer mixture (first primers targeting three SARS-Cov-2 genes were mixed as first primer set) for 10 min and second RPA primer mixture (second primers and probes targeting three SARS-Cov-2 were mixed as second primer set) for another 10 min. The sequence and concentration of each target used in multiple reaction were identical to standard nested RPA 2.0 except that the reverse-primers targeting E gene, N gene and ORF1ab gene in second primer set were labeled with digoxin, TAMRA and Biotin respectively that were captured correspondingly by antibodies located on the LFA strip. The copy number used in this test was labeled on the bottom of each picture.

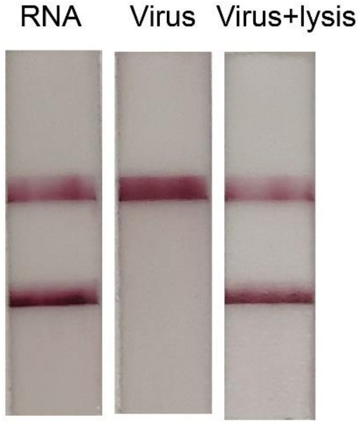

Figure S12. Lysis buffer improves detection sensitivity for virus by nucleic acid release.

Samples with 10 copies purified RNA, intact virus and virus treated with lysis buffer were subjected to nested RPA 2.0 reaction respectively. The results shown on LFA strip suggest the improvement of sensitivity by lysis buffer on virus detection.

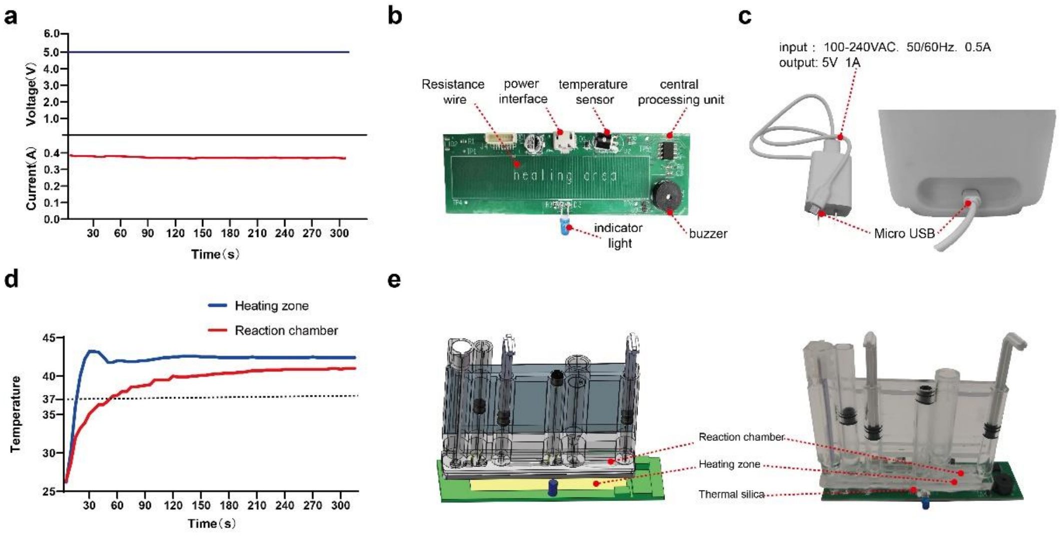

Figure S13. Cartridge heating system. a) Characterization of the PCB board current and voltage. After the BINAS system is powered on, the system voltage is 5V, the current is about 0.4A, and the power is about 2W. b) Photograph of the PCB board. This PCB circuit board is controlled by an 8-bit central processing unit. The reaction area is heated by a resistance wire, and the heating temperature is monitored by a temperature sensor, and the buzzer and indicator light are used to feedback the heating state. c) Connection of power supply and BINAS. The power interface of BINAS is Micro USB, which only needs a power source similar to a mobile phone charger. d). Temperature calibration of the BINAS. The set temperature (blue) was calibrated according to the actual temperature in the amplification chamber (red). During the RPA amplification reaction, the temperature in the reaction chamber is maintained at around 41°C. e) Connection between the microfluidic cartridge and the heating area of the PCB board. To ensure temperature accuracy, apply thermally conductive silicone between the microfluidic cartridge and the heated area to remove the gap.

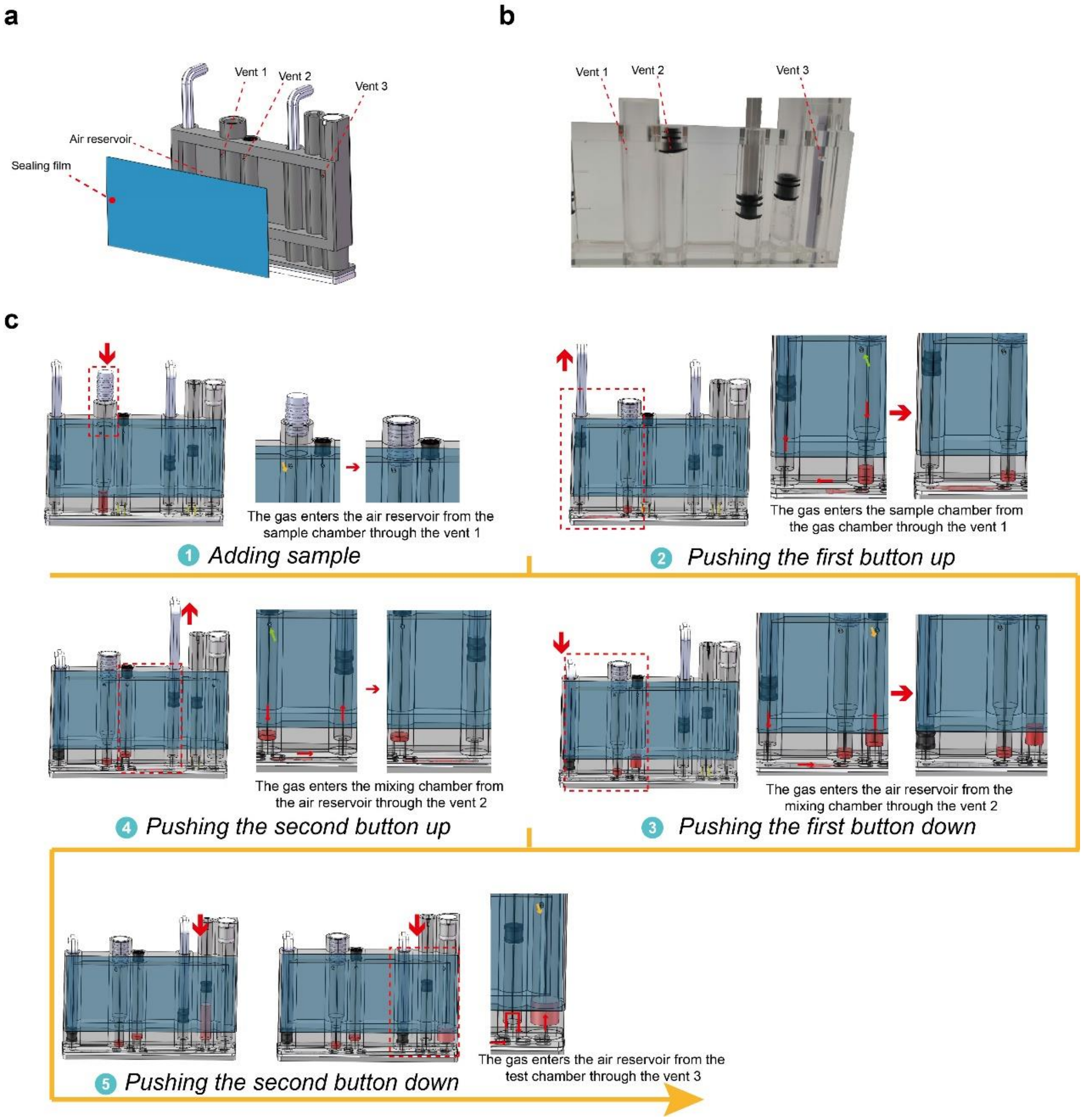

Figure S14. The air circulation system in the cartridge. a) Schematic of the air circulation system showing components in an exploded view. The system consists of three vents, an air reservoir and a sealing membrane. b) Photograph of the air circulation system. c) Overall the air circulation system workflow. (1) Adding sample: The gas enters the air reservoir from the sample chamber through vent 1. (2) Pushing the first button up: The gas enters the sample chamber from the gas chamber through vent 1. (3) Pushing the first button down: The gas enters the air reservoir from the mixing chamber through vent 2. (4) Pushing the second button up: The gas enters the mixing chamber from the air reservoir through vent 2. (5) Pushing the second button down: The gas enters the air reservoir from the test chamber through vent 3.

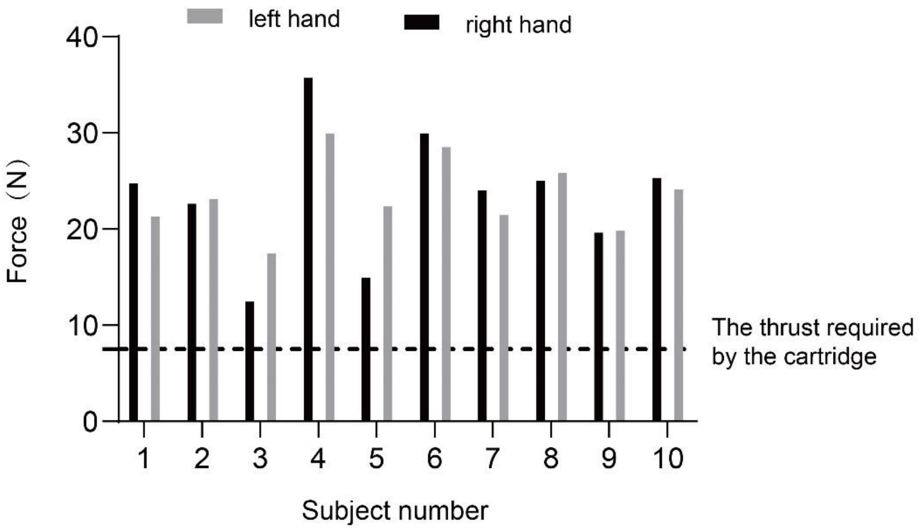

Figure S15. Thumb thrust test on 10 volunteers under relaxed conditions. In the experiment, a dynamometer was used to test the thumb thrust of 10 volunteers, and the test results were all greater than the minimum thrust required by the cartridge drive.

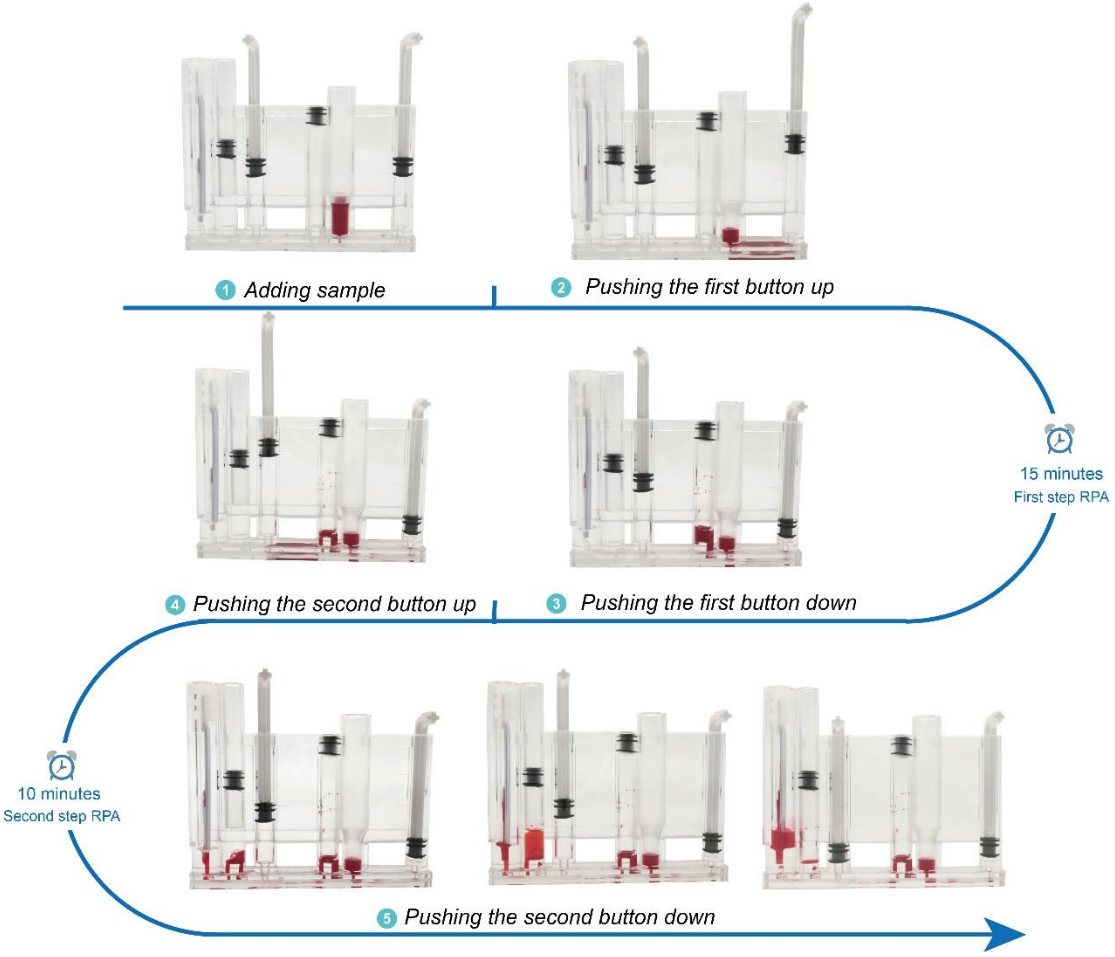

Figure S16. Overall BINAS workflow. The physical diagram of the fluid operation corresponds to the flow in Fig. 3c. The red dye is used in BINAS to visualize the movement of liquid in the microfluidic cartridge.

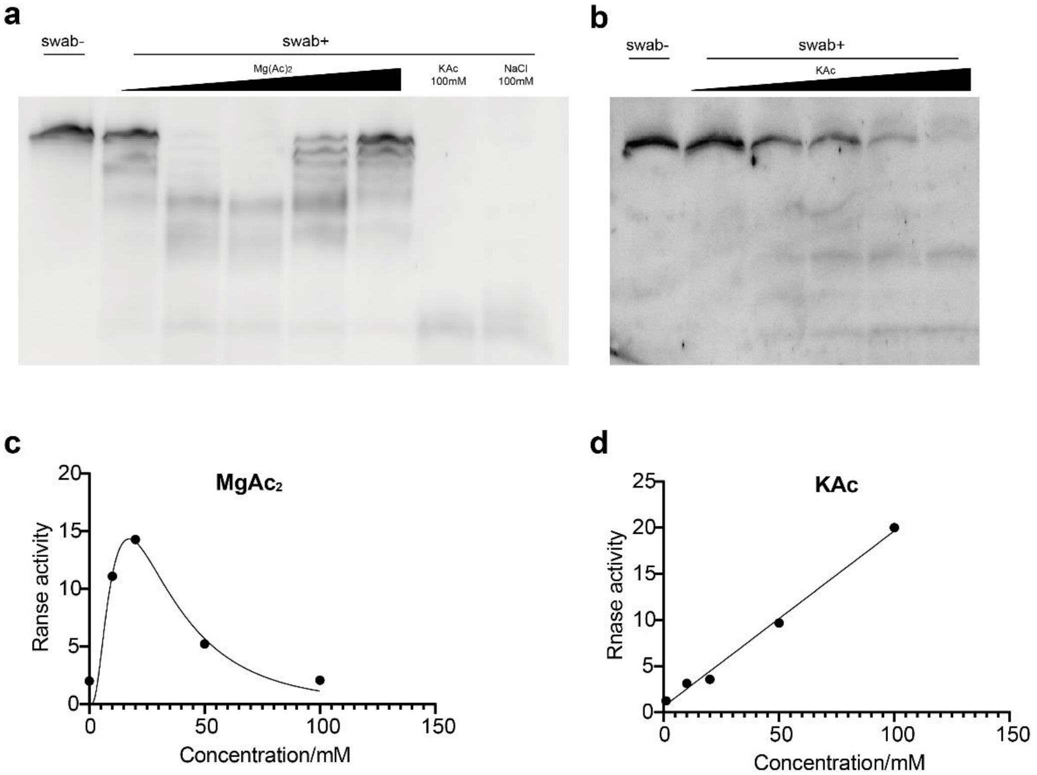

Figure S17 RNase activity is activated by positive ions in a concentration-dependent manner.

RNA standards were subjected to swab buffer with ascending concentration of MgAc2 or KAc for 5 min followed by PAGE electrophoresis for RNase activity assay. The relative RNase activity was evaluated following the equation that the gray value of intact RNA was divided by the gray value of residual RNA. The row data was shown in a, b and the calculated RNase activity data was shown in c, d.

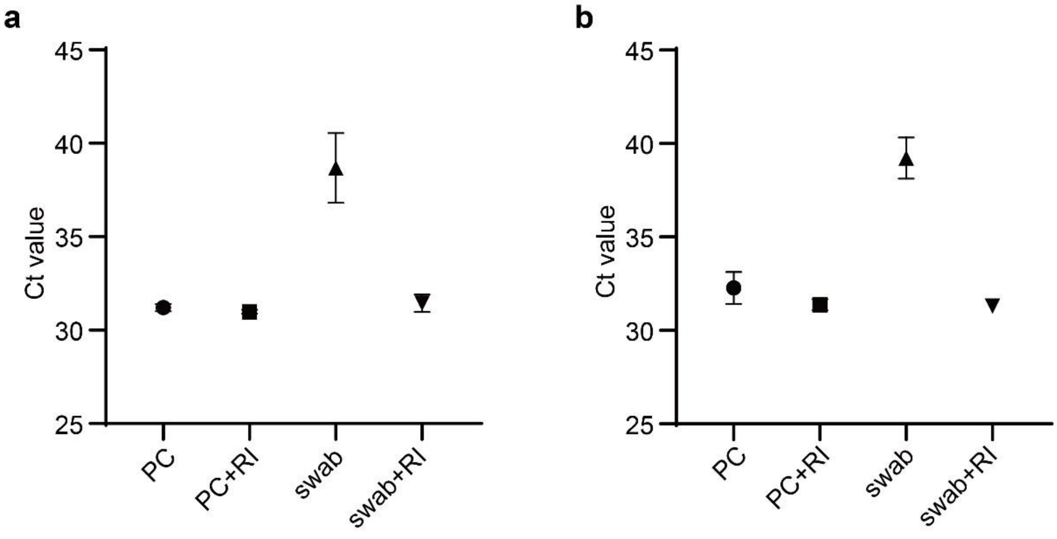

Figure S18. RNase Inhibitor was able to restore most detection signal.

a) RNA templates were incubated in lysis buffer (PC group) and swab buffer (swab group) with or without RNase Inhibitor (RI) for 5 min followed by quantitative analysis with RT-qPCR. The Ct value was decreased with RI addition indicating the inhibitory effect of RI to RNase in swab samples. b) The samples treated from a) were re-transcript into DNA by reverse transcriptase. The DNA products were quantitatively analyzed by qPCR. The results showed that the RT products were recovered to PC level when RNase was inhibited by RI indicating that the RT enzyme was not inhibited in swab buffer. Data are presented in term of the mean ± standard deviation with n=3

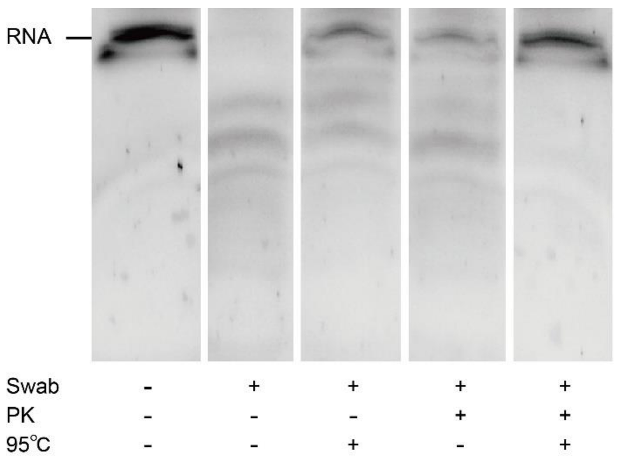

Figure S19. RNase was totally digested by PK at high temperature.

Swab samples digested by proteinase K (PK) at high (95℃+) or low (95℃-) were subjected to RNase activity assay by RNA-PAGE electrophoresis. Lane 1: intact RNA samples; lane 2: RNA samples were incubated for 5min with swab buffer; lane 3: RNA samples incubated for 5min with swab buffers pre-treated by heating for 5min; lane 4: RNA samples were incubated for 5min with swab buffer pre-treated by PK for 5min at room temperature; lane 5: RNA samples was incubated for 5min in swab buffer pre-treated by PK for 5min at 95 degrees. The result showed in RNA-PAGE indicated that RNase in swab buffer was degraded by treatment of proteinase K at 95 degrees.

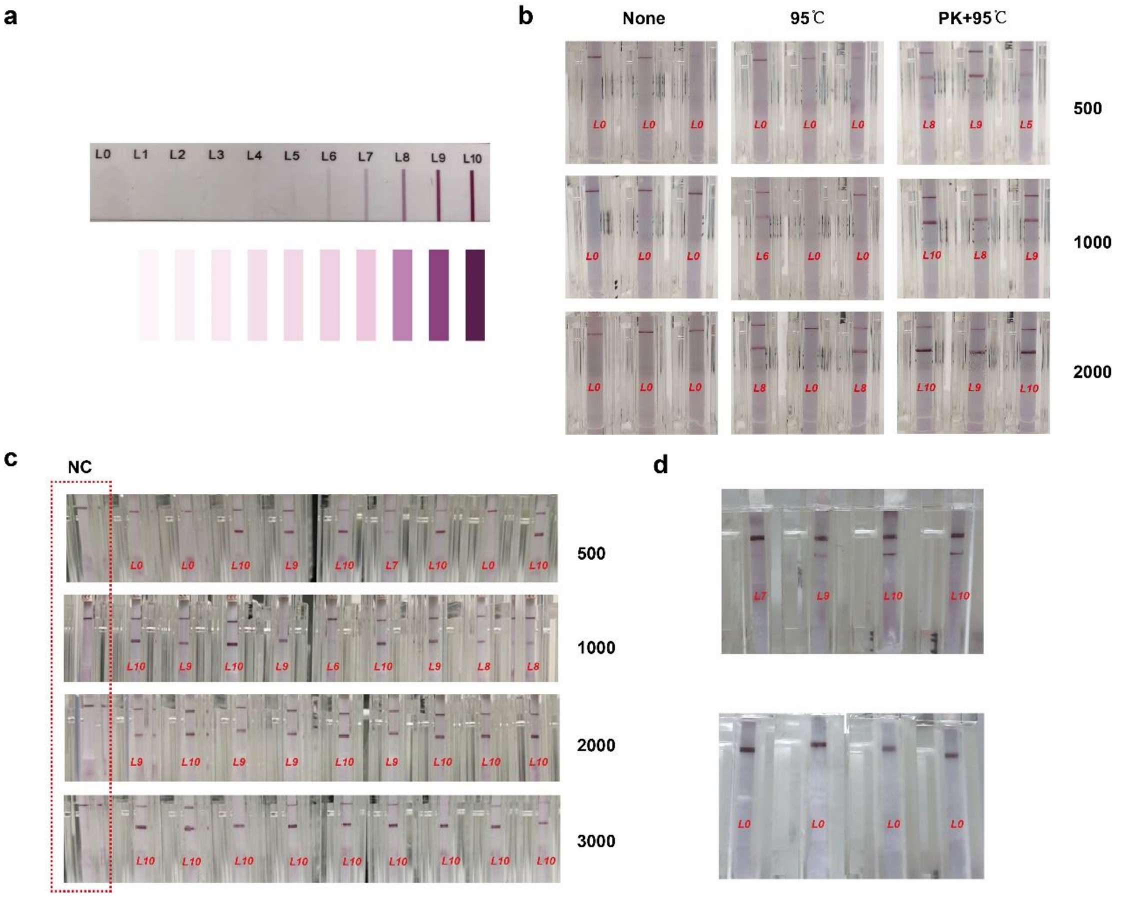

Figure S20. Detection of simulated samples and clinical samples. a) Test strip color grade. According to the depth of the color band of the test strip, it can be divided into 11 grades from L0 to L10. b) Photo of detection sensitivity of multi-person mixed samples Under different sample pretreatment conditions, corresponding to Figure 5d left. c) Photo of detection sensitivity of single samples in the case of sample pretreatment with proteinase K heated at 95°C, corresponding to Figure 5d right. d) Photo of test results of clinical samples. Top: positive sample test results; bottom: negative sample test results.

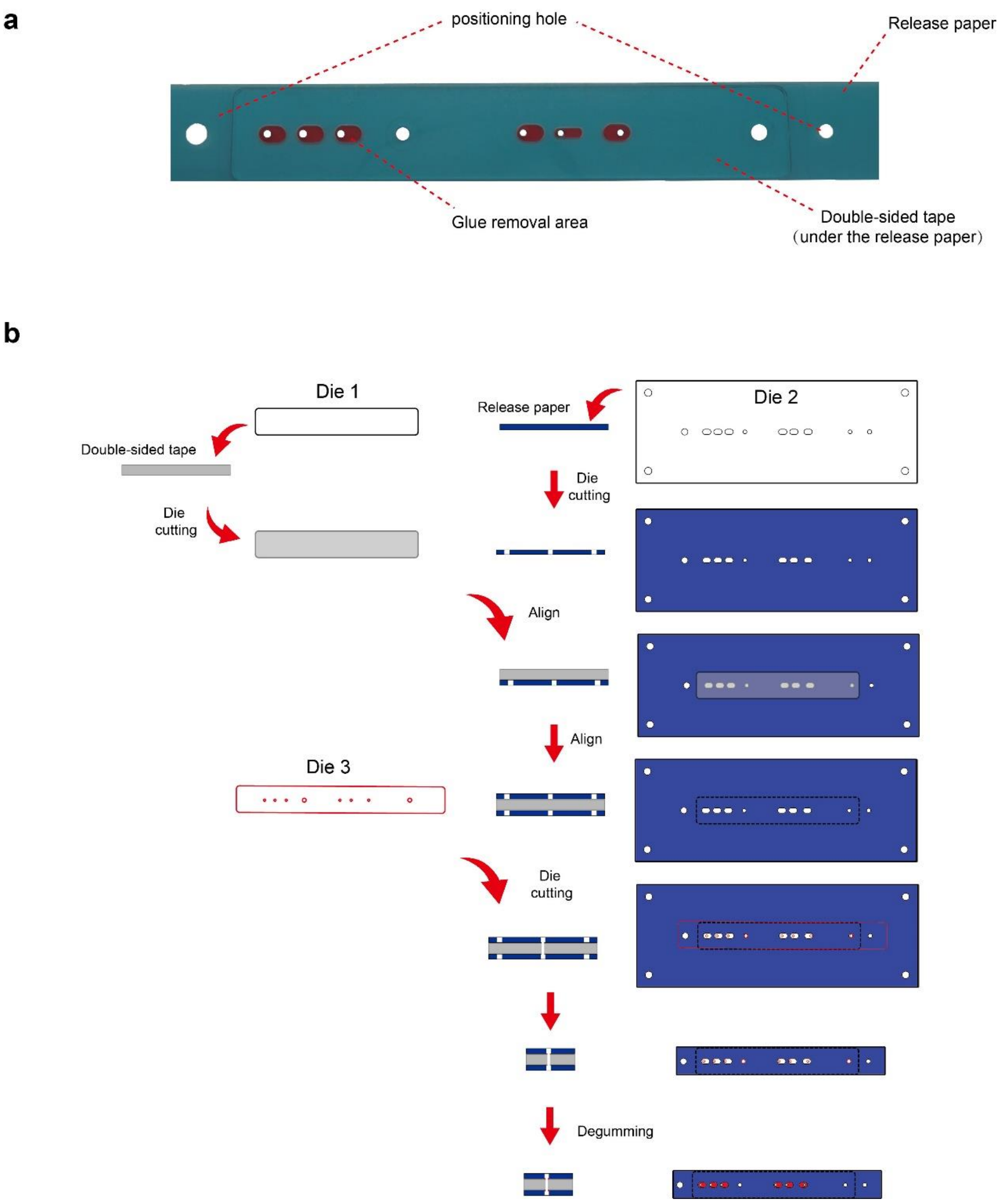

Figure S21. Production of double-sided tape. a) Photograph of the DS tape. The release paper protects the adhesive area on both sides of the double-sided tape. Positioning holes are used for alignment bonding during chip processing. b) The non-adhesive patterning procedure of the DS tape. First, pieces of release paper were cut by the die to form the pattern we wanted. Then, a DS tape piece was covered with the patterned release paper as masks from both sides. The second die punched holes in the tape's corresponding position to form the valves. Last, Talc powder was smeared on the surface of the release paper. Where the release paper did not cover the double-sided tape, the talcum powder contacted the adhesive, thereby removing the adhesiveness of the tape, and the excess talcum powder was blown away by nitrogen.
